# Phytomedicines in preclinical and clinical research on antipsychotics-induced EPS/EPS-like behaviors: therapeutic effects and neurobiological mechanisms

**DOI:** 10.3389/fpsyt.2026.1836674

**Published:** 2026-06-19

**Authors:** Ziwen Wang, Huicong Shi, Chengbin Guan, Xin-Min Li, Haiyun Xu

**Affiliations:** 1 The Affiliated Wenzhou Kangning Hospital, School of Mental Health, Wenzhou Medical University, Wenzhou, China; 2 Department of Rehabilitation Medicine, The Second Affiliated Hospital of Wenzhou Medical University, Wenzhou, China; 3 Department of Psychiatry, Faculty of Medicine & Dentistry, University of Alberta, Edmonton, AB, Canada

**Keywords:** antioxidant, antipsychotics, dopamine receptors, extrapyramidal syndrome, phytomedicines, schizophrenia

## Abstract

Extrapyramidal syndrome (EPS) refers to a group of movement disorders resulting from dysfunction of the extrapyramidal system (motor pathways outside the pyramidal tract). Among the various pathogenic factors of EPS, chronic or long-term use of antipsychotic drugs (APDs) is worth special note. This article aims to provide a comprehensive review on the application of phytomedicines in preventing and treating the APDs-induced EPS or EPS-like behaviors by summarizing their therapeutic effects and outlining the underlying neurobiological mechanisms. An advanced search was done using the data bases PubMed, Web of Science, Google Scholar, and CNKI, employing the terms “antipsychotics, “ “dopamine, “ “extrapyramidal syndrome, “ “movement disorder”, “herbal, “ “haloperidol, “ “phytomedicine”, and “traditional Chinese medicine”. The Boolean operators “AND”, “OR” were used to combine some of the above terms in the literature search. Except for a few negative reports, the extant clinical research reported beneficial effects of herb medicines on EPS in patients with schizophrenia. Most of all the research reviewed in this article are animal studies, in which herbal medicines prevented or improved the APDs-induced EPS-like behaviors in animal models of EPS. The neurobiological mechanisms underlying the observed therapeutic effects involve regulating monoaminergic neurotransmission, alleviating oxidative stress, maintaining mitochondrial function, suppressing neuroinflammation, and modulating the KEAP1/Nrf2 pathway, which regulates redox homeostasis and mitochondrial function and plays a crucial role in regulating neuroinflammation. The results of existing research support the combination of herbal medicines with antipsychotic drugs for the prevention and treatment of EPS in patients with schizophrenia.

## Introduction

Extrapyramidal syndrome (EPS) refers to a group of movement disorders resulting from dysfunction of the extrapyramidal system (motor pathways outside the pyramidal tract). The primary physiological functions of this system include regulating muscle tone, maintaining body posture, and coordinating voluntary movements (1). Consequently, EPS may manifest as any of the following clinical phenotypes: 1) acute dystonia, characterized by involuntary abnormal movements such as oculogyric crisis, torticollis, neck hyperextension, and facial grimacing; 2) akathisia featuring with uncontrollable subjective anxiety/restlessness and objective pacing behaviors; 3) pseudoparkinsonism, presenting with the classic triad of bradykinesia, catalepsy, and tremor; and 4) tardive dyskinesia (TD), involving abnormal involuntary irregular movements of the orofacial region and trunk, typically including vacuous chewing movements (VCMs), tongue protrusions (TPs), and facial jerkings (FJs) (2, 3). EPS commonly occurs in patients with Parkinson’s disease (PD), chorea, Huntington’s disease (HD), and Tourette syndrome (TS), with etiological factors encompassing brain injury, infections, genetic predisposition, neurodegenerative processes, and medication side effects.

As a medication side effect, EPS induced by chronic or long-term use of antipsychotic drugs (APDs) warrants particular attention. Almost all APDs exert their therapeutic effects by binding to dopamine receptors (DRs). When DRs in the nigrostriatal pathway are inhibited, striatal dopamine (DA) function weakens while acetylcholine (ACh) activity becomes relatively enhanced, ultimately leading to dystonia (4). Among the four EPS phenotypes mentioned above, acute muscle tone disorder appears earliest, usually within a few days to a week after the onset of DR blockade, with 90% of cases occurring within the first three days of treatment (5); akathisia usually develops within two weeks after starting medication (6); APDs-induced pseudoparkinsonism generally appears within one month of treatment, with 90% of cases occurring within three months (7). These three phenotypes are collectively termed early-onset movement disorders, whereas TD typically emerges after three months of antipsychotic treatment (8), represents the most severe adverse effect of APDs (9).

EPS significantly increases patients’ feelings of stigma and psychological distress while reducing medication adherence (10), often leading to voluntary discontinuation of treatment and subsequent disease relapse, making full recovery difficult to achieve (11–13). Even when managed under medical supervision through drug withdrawal or alternative therapies including anticholinergic injections or substitution with second-generation antipsychotics (SGAs), complete prevention of EPS remains unattainable. This is because long-term use of SGAs can also induce EPS, albeit with lower incidences (14). Treatment discontinuation may exacerbate the underlying condition (15), while anticholinergic medications themselves carry substantial side effect burdens. Consequently, the prevention and management of EPS have emerged as a major challenge requiring urgent solutions in psychiatric practice.

Notably, many preclinical and clinical studies have explored the application of phytomedicines in the prevention and treatment of EPS. Certain herbal medicines, such as ginkgo biloba (16–18), curcumin (19–21), and quercetin (22, 23), have shown therapeutic efficacy in APDs-induced EPS in humans and EPS-like behaviors in preclinical research. Research has also reported that naringin and betaine ameliorated haloperidol-induced orofacial dyskinesia (24, 25), while hesperidin and nardostachys jatamansi reversed EPS-associated catalepsy (26, 27). In addition, Ilex paraguariensis showed potential to improve memory impairment in rats exposed to APDs (28).

To provide a scope review on the applications of phytomedicines in preventing and treating EPS induced by APDs, we conducted a comprehensive literature search and analyzed the data from clinical and preclinical research on this topic. Here we summarized the therapeutic effects of 5 herb formulas, 5 individual herbs/herbal ingredients, 13 herb extracts, and 34 phytochemicals on APDs-induced EPS or EPS-like behaviors. Moreover, we outlined the neurobiological mechanisms underlying the therapeutic effects of the phytomedicines on APDs-induced EPS or EPS-like behaviors.

## Methodology

### Search strategy

This narrative review adopted a structured, though not systematic, approach to identify relevant literature. Specifically, studies published between 1989 and 2025 in Chinese and English were searched in PubMed, Web of Science, GeenMedical, Google Scholar, and CNKI. The search strategy employed the following key terms: “antipsychotics, “ “dopamine, “ “extrapyramidal syndrome, “ “movement disorder”, “herbal, “ “haloperidol, “ “mitochondrial dysfunction, “ “neuroinflammation, “ “oxidative stress, “ “phytomedicine”, and “traditional Chinese medicine”. The Boolean operators “AND”, “OR” were used to combine some of the above terms in the literature search.

### Inclusion and exclusion criteria

We considered eligible English and Chinese language articles published in peer-reviewed journals, exploring potential protective effects of phytomedicines on APD-induced EPS in schizophrenia patients and preclinical research, the involvement of mitochondrial dysregulation in schizophrenia, and their implication in antipsychotic treatment. Clinical studies exploring the antioxidant effects of phytomedicines in patients with schizophrenia were included. Original clinical and preclinical research studies and reviews were included. Conference abstracts, commentaries, and letters to the editor were excluded.

## Therapeutic effects of herb formulas on the APDS-induced EPS/EPS-like behaviors and neurobiological changes (**Table 1**)

**Table 1:** Therapeutic effects of herb formulas on EPS/EPS-like behaviors and the underlying neurobiological changes.

Herb formulas	Components of the formulas	Subjects	Therapeutic effects	Neurobiological changes	References
Wendan decoction (WDD)	Rhizoma pinellie, Bambusae caulis in taenian, Pericarpium citri reticulatae, Fructus aurantii immaturus, Rhizoma zingiberis, Radix glycyrrhizae	SCZ patients; MK-801treated rats	Mental state↑, EPS↓ PANSS↓, BPRS↓; Cognitive function↑	IL-2↓, oxygen free radical↓, DA↑	(29) (30) (31)
Kamishoyosan (KSS)	Glycyrrhizae radix, Angelicae gigantis radix, Bupleuri radix, Gardeniae fructus, Zingiberis rhizoma recens, Cortex moutan radicis, Atractylodis rhizoma alba, Hoelen, Paeoniae radix, Menthae herba	SCZ patients	AIMS↓, Tremor↓	/	(32) (33),
Yokukansan (YKS)	Angelica acutiloba L, Atractylodes lancea DC, Bupleurum falcatum L, Poria cocos Wolf, Glycyrrhiza uralensis, Cnidium officinale Makino, Uncaria rhynchophylla Schreb	SCZ patients Haloperidol-treated Wistar rats	Psychotic symptoms↓ PANSS↓, VCMs↓	Glutamate ↓ GLT-1 mRNA↑	(35, 36) (37)
SG-Tang	Radix paeoniae (peony) Radix glycyrrhizae (licorice)	SCZ patient	EPS↓		(38)
NR-ANX-C (polyherbal formulation)	Withania somnifera, Ocimum sanctum, Camellia sinensis, Triphala, Shilajit	Haloperidol treated mice	Cataleptic scores↓	Normalized SOD activity	(42)

AIMS, abnormal involuntary movement scale; BPRS, brief psychiatric rating Scale; DA, dopamine; EPS, extrapyramidal symptoms; GLT-1, glutamate transporter-1; PANSS, positive and negative syndrome scale; SCZ, Schizophrenia; VCM, vacuous chewing movements.

A herbal formula in traditional Chinese medicine (TCM) is composed of multiple herbs combined in specific proportions. With a long history of clinical use, herb formulas in TCM have demonstrated well-documented efficacy in treating schizophrenia, idiopathic PD, Alzheimer’s disease (AD), and depression. Below, we summarize representative clinical and preclinical studies on the combination of herb formulas and APDs for the management of EPS/EPS-like behaviors.

### Wendan decoction

WDD typically contains Rhizoma pinellie, Bambusae caulis in taenian, Pericarpium citri reticulatae, Fructus aurantii immaturus, Rhizoma zingiberis, and Radix glycyrrhizae. The formula can be modified by adjusting individual herb dosages to target specific symptoms. A recent intervention review published in Cochrane Library included 15 randomized trials (1437 participants) of WDD for schizophrenia. The data showed that WDD improved the short-term global state of participants compared with placebo or no treatment. And WDD showed good features and potentials in reducing the APDs-induced EPS, while limited evidence suggested that WDD may have some antipsychotic effects as measured on global and mental state (29). In another systematic review of randomized controlled trials involving 1, 174 schizophrenia patients, the pooled results showed that WDD combined with APDs were more effective in clinical comprehensive effect as measured by Positive and Negative Syndrome Scale (PANSS) scores and Brief Psychiatric Rating Scale (BPRS) scores compared with APDs alone. The side effects were significantly reduced in the intervention group compared with the control group (30). In an earlier animal study, WDD improved learning and memory deficits in a rat model of schizophrenia (31).

### Kamishoyosan

This formula consists of the extracts of *Glycyrrhizae radix, Angelicae gigantis radix, Bupleuri radix, Gardeniae fructus, Zingiberis rhizoma recens, Cortex moutan radicis, Atractylodis rhizoma alba, Hoelen, Paeoniae radix*, and *Menthae herba*. It has been traditionally prescribed for patients with neuropsychiatric symptoms in Japan. In an earlier clinical study, KSS was effective against the tremor in patients with parkinsonism, and the tremor did not worsen in any of the patients, and none complained of side-effects (32). Later on, a 16-week open clinical trial evaluated the clinical effectiveness of KSS for adjunctive treatment of TD in patients with schizophrenia. It reported meaningful reduction in total abnormal involuntary movement scale (AIMS) scores in the TD group treated with KSS at 4, 8, and 16 weeks of treatment (33).

### Yokukansan

Yokukansan (Yi-gan san; TJ-54; YKS) is a traditional herbal medicine with evident therapeutic effect for neuropsychiatric disorders. It contains a mixture of seven dried herbs, including *Angelica acutiloba L., Atractylodes lancea DC., Bupleurum falcatum L., Poria cocos Wolf., Glycyrrhiza uralensis, Cnidium officinale Makino*, and *Uncaria rhynchophylla Schreb* in a ratio of 3:4:2:4:1.5:3:3, respectively (34). In an open-label study with very-late-onset schizophrenia patients, a highly significant improvement on all measures of psychotic symptomatology was observed in all patients (35). In a randomized, multicenter, double-blind, placebo-controlled study with one hundred twenty antipsychotic-treated inpatients from 34 psychiatric hospitals in Japan, schizophrenia patients were randomized to adjuvant treatment with YKS 7.5 g/day or placebo. During a 4-week follow-up, psychopathology was assessed using the PANSS with five factors, depression/anxiety, cognition, positive, and negative. Compared to the placebo group, the YKS group showed statistically significant improvements in the PANSS excitement/hostility factor scores (36). Animal studies revealed dose-dependent reductions in haloperidol-induced VCMs after 21-day YKS administration to Wistar rats. The formula normalized striatal glutamate elevation and GLT-1 mRNA downregulation caused by haloperidol, suggesting modulation of extracellular glutamate and GLT-1 expression as potential mechanisms (37).

### Shaoyao Gancao Tang

SG-tang (Jakyak-Gamcho decoction in Korean; Shakuyaku-Kanzo-To in Japanese) is a formulated traditional Chinese herbal medicine made of *P. lactiflora* and *G. uralensis* at a 1:1 ratio. In a randomized, open-label study with twenty Japanese patients with psychiatric disorders and experiencing EPS during antipsychotic treatment, Shakuyaku-Kanzo-To improved EPS, with no significant changes in psychiatric symptoms, plasma homovanillic acid (HVA) levels, or serum prolactin levels (38). Later on, SG-Tang displayed neuroprotection by exerting antioxidative and anti-inflammatory activities to suppress neuronal apoptosis in human tau cell models (39). In addition, SG-Tang reduced aggregation and exerts neuroprotection in spinocerebellar ataxia type 17 (SCA17) cell and mouse models (40).

### NR-ANX-C

This polyherbal formula contains bioactives of *Withania somnifera, Ocimum sanctum, Camellia sinensis, triphala* and *shilajit*. The constituents of NR-ANX-C have been shown to effect on the monoaminergic and GABAergic neuronal systems (41) in the brain. In an animal study with male albino mice treated with haloperidol, NR-ANX-C (10, 25 and 50 mg/kg) and scopolamine (1 mg/kg) were compared in their anticataleptic efficacy. Significant reduction in the cataleptic scores was observed in all NR-ANX-C treated mice and maximum reduction was found in the mice treated with the highest dose (25 mg/kg). Moreover, significant reduction in superoxide dismutase (SOD) activity was observed in NR-ANX-C (25 and 50 mg/kg) treated mice, suggesting an antioxidant-mediated neuroprotection (42).

## Therapeutic effects of individual herbs/herbal ingredients on the APDS-induced EPS/EPS-like behaviors and neurobiological changes (**Table 2**)

**Table 2:** Therapeutic effects of individual herbs on EPS/EPS-like behaviors and the underlying neurobiological changes.

Individual herbs	Active ingredients	Subjects	Therapeutic effects	Neurobiological changes	References
M. pruriens	DA 5-HT	Patients with PD Haloperidol-treated Albino rats	PD symptoms↓ VCMs↓	Hydroxyl radical↓	(44) (46)
Harpagophytum Procumbens (HP)	Harpagoside, Phenolic compounds	Fluphenazine treated rats, Amphetamine treated rats	VCMs↓, Crossings↑, Rearings↑, Stereotypies↓, Working memory↑	CAT↑, ROS↓	(48) (49)
Spirulina maxima	Proteins, Vitamins, Essential aminoacids, Minerals, Essential fatty acids	Haloperidol treated rats, 6-OHDA treated rats	VCMs↓, Rotations↓, Locomotion↑, Rest↓, Path traveled↑, Stereotype↑, Rearings↑, Muscle coordination↑	SOD↑, GSH↑, CAT↑, VitC↑, LPO↓, GSH↑, DA↑, DOPAC↑, Nitrite↑, MDA↓, TH↑, DAT↑, iNOS↓, COX-2↓	(50) (51) (52)
Nigella sativa Oil	Gallic acid, Catechin, Chlorogenic acid, Caffeic acid, Orientin, Rutin, Quercitrin, Quercetin, Luteolin	Haloperidol treated rats	Motor coordination↑, VCMs↓	Activated microglia↓, Neuronal density↑, GSH↑, GST↑, CAT↑, LDH↑, MDA↓, AChE↓, BChE↓, MAO↓	(54) (55)
Rice bran oil	Tocopherol, Tocotrienol, γ-oryzanol, β-sitosterol, Unesterified fatty acids	Albino Wistar rats treated with haloperidol	VCMs↓, Exploratory activity↑, Open field activity↑, Home cage activity↑, Motor coordination↑	MDA↓, H_2_O_2_↓, DA↑, HVA↑, DOPAC↑, SOD↑, CAT↑, GPx↑	(56) (57)

5-HT, serotonin; AChE, acetylcholinesterase; BChE, butyrylcholinesterase; CAT, catalase; COX-2, cyclooxygenase-2; DA, dopamine; DAT, dopamine transporter; DOPAC, dihydroxyphenylacetic acid; GPx, glutathione peroxidase; GSH, glutathione; GST, glutathione-S-transferase; HVA, homovanillic acid; iNOS, inducible nitric oxide synthase; LDH, lactate dehydrogenase; LPO, lipid peroxidation; MAO, monoamine oxidase; MDA, malondialdehyde; PD, Parkinson’s disease; ROS, reactive oxygen species; SOD, superoxide dismutase; TH, tyrosine hydroxylase; VCM, vacuous chewing movements.

### Mucuna pruriens

The medicinal plant *M. pruriens* (Fabaceae) is widely known for its anti-oxidative and anti-inflammatory properties. It has long been used to treat PD in Ayurvedic medicine (43, 44). In an earlier animal study, M. pruriens (100 mg/kg) showed significant anticataleptic and antiepileptic activity in albino rats (45). In another animal study, M. pruriens attenuated haloperidol-induced VCM and orofacial bursts while inhibited hydroxyl radical generation, suggesting that this plant medicine exerts neuroleptic-induced TD by virtue of its free radical scavenging activity (46). In patients with PD, M. pruriens prolonged the onset time 2-fold compared with an equivalent levodopa or carbidopa, and the time to the onset of therapeutic effects was shorter in the M. pruriens group than in the levodopa group, suggesting it works faster and longer than levodopa (47).

### Harpagophytum procumbens

This herbal medicine has been shown to exhibit anti-inflammatory and antioxidant effects. In an animal study, EAF-HP inhibited the fluphenazine-induced VCMs and oxidative damage as indicated by changes in catalase (CAT) activity and reactive oxygen species (ROS) levels in the cortex and striatum of the rat brain (48). In mice, the pre-treatment with EAF-HP for 7 days protected against positive and cognitive symptoms, as well as stereotypies induced by amphetamine (49).

### Spirulina maxima

*Spirulina maxima* is a filamentous cyanobacterium (blue-green algae) rich in proteins and antioxidants. In an animal model of EPS, Spirulina supplementation at a dose of 180 mg/kg significantly improved enzymatic and nonenzymatic antioxidants and decreased the haloperidol-induced TD (50). Related to this finding, both pre- and post-administration of spirulina extract showed a significant protective effects on the 6-hydroxydopamine (6-OHDA)-induced abnormal behaviors and high levels of oxidant-generating enzymes in rats (51). Compared with untreated 6-OHDA injured rats, spirulina has a neuroprotective effect in the 6-OHDA-induced hemiparkinsonism, manifested by reduced rotational behavior induced by apomorphine. The treatment also recovered the drastic decrease in tyrosine hydroxylase (TH) and dopamine transporter (DAT) immunoreactivities in the rat brain (52).

### Nigella sativa oil

Derived from Nigella sativa seeds, this oil contains multiple bioactive components, particularly thymoquinone, which exhibits immunomodulatory and antioxidant protective properties (53). Experimental studies have demonstrated its ability to reverse haloperidol-induced EPS-like behaviors. Further biological investigations revealed that the oil reduces activated microglial cells and restores average neuronal density (54). Additionally, it significantly enhances antioxidant capacity by increasing glutathione (GSH), Glutathione-S-transferase (GST), and lactate dehydrogenase (LDH) levels while decreasing malondialdehyde (MDA) content. Moreover, the oil inhibited the enzymatic activities of acetylcholinesterase (AChE), butyrylcholinesterase (BChE), 5’-nucleotidase, and monoamine oxidase (MAO), while preventing vacuolization and loss of substantia nigra neurons (55).

### Rice bran oil

With abundant tocopherols, tocotrienols, γ-oryzanol, and polyphenolic compounds, this oil exhibits diverse pharmacological properties including nootropic, anxiolytic, and anti-inflammatory effects. Interestingly, administration of rice bran oil by oral tubes at a dose of 0.4 mL/day prevented the induction of haloperidol-elicited VCMs and impairment of motor coordination. This protective effect was at least in part due to its antioxidant effects given that the rice ban oil normalized SOD activity, reduced hydrogen peroxide (H_2_O_2_) production, and increased the activity of CAT, as well as normalized glutathione peroxidase (GPx) activity in the rat striatum (56, 57).

## Therapeutic effects of herb methanol extracts on the APDS-induced EPS-like behaviors and neurobiological changes (**Table 3**)

**Table 3:** Therapeutic effects of methanol extracts of herbs on EPS-like behaviors and the underlying neurobiological changes.

Herbs	Active ingredients	Subjects	Therapeutic effects	Neurobiological changes	References
Morus alba (MEMA)	Carotene, Vitamin B1, Folic acid, Folinic acid, Isoquercetin, Quercetin, Tannins, Favonoids, Saponins	Haloperidoltreated rats Amphetamine-treated Swiss mice	VCMs↓, TPs↓ Stereotyped behavior↓	LPO↓, nitrite↓, GSH ↑, SOD↑, CAT↑	(60) (59)
Cucurbita pepo seeds (MECP)	Gallic acid, Ferulic acid, Caffeic acid, Chlorogenic acid	Haloperidol treated rats	Cataleptic scores↓, Motor dysfunction and anxiety↓	NT ↓, MDA↓, nitrite ↓ CAT↑, GSH↑, SOD↑ DA↑, 5-HT↑, NE↑ TNF-α↓, IL-1β↓	(62)
Myrica esculenta (MEME)	Flavonoids, Arylheptanoids, Phenolic compounds	Haloperidol treated rats	Catalepsy scores↓, FJs↓	MDA↓, CAT↑, GSH↑, SOD↑	(64)

5-HT, serotonin; CAT, catalase; DA, dopamine; FJs, facial jerkings; GSH, glutathione; IL-1β, interleukin-1β, LPO, lipid peroxidation; MDA, malondialdehyde; NE, norepinephrine; NT, neurofibrillary tangle; SOD, superoxide dismutase; TNF-α, tumor necrosis factor-α; TPs, tongue protrusions; VCMs, vacuous chewing movements.

### Methanol extract of *Morus alba*

In traditional Chinese and Indian medicine, the leaves of the mulberry tree have been used as an astringent, anthelmintic, antibacterial, purgative, diaphoretic, diuretic, analgesic, antiasthmatic, antirheumatic, antitussive, emolient, expectorant, hepato-protective, hypoglycemic, and brain tonic. In addition, they have shown antiviral and antimicrobial activity (58). MEMA contains carotene, vitamin B1, folic acid, folinic acid, isoquercetin, quercetin, tannins, favonoids, and saponins, which act as a good source of natural antioxidants. In Swiss mice, methanolic extract of *Morus alba* (50, 100 and 200 mg/kg, i.p.) significantly blocked amphetamine-induced stereotyped behavior in the animals (59). In an animal model of TD, MEMA ameliorated the haloperidol-induced VCMs and TPs while normalizing levels of lipid peroxidation, nitrite, SOD, and CAT, in comparison to the control group (60).

### Methanol extract of *Cucurbita pepo*

*Cucurbita pepo* (*C. pepo*) is cultivated and used traditionally as vegetable as well as medicine in different parts of the world. *C. pepo* is rich in antioxidants such as flavonoids and phenolic acids, along with small amounts of vitamin E and carotenoids. It’s thought that the high levels of antioxidants in *C*. *pepo* are partly responsible for its positive effects on health (61). In a rat model of PD, *C. pepo* effectively reduced the score of rigidity and ameliorated haloperidol-induced motor dysfunction, particularly the decreased locomotion distance. In the meanwhile, *C. pepo* normalized oxidative stress biomarkers by decreasing MDA and nitrite levels while enhancing CAT, SOD and GSH activities. Moreover, *C. pepo* restored neurotransmitter homeostasis (DA, serotonin, and norepinephrine) while reducing α-synuclein misfolding and down-regulating pro-inflammatory cytokines (tumor necrosis factor-α, TNF-α, and interleukin-1β, IL-1β) (62). Related to these findings, a recent animal study reported that petroleum ether extract of *C. pepo* and hydroethanolic extract of *C. pepo* significantly attenuated the behavioral changes including hyperalgesia, allodynia and motor nerve conduction velocity linked to diabetic neuropathy. Moreover, the oxidative stress and levels of TNF-α, TGF-β and IL-1β were found to be significantly attenuated in the *C. pepo* treated animals (63).

### Methanol extract of *Myrica esculenta*

*Myrica esculenta* (*M. esculenta*) is a notable therapeutic plant widely utilized in Indian system of medicine. Qualitative phytochemical screening of *M. esculenta* leaves showed the presence of alkaloids, sugars, phenolic compounds, flavonoids, glycosides, and tannins (64). In a rat model of PD induced by 1 mg/kg of haloperidol over one week, the MEME treatment effectively prevented haloperidol-induced increases in catalepsy and FJs while protecting brain neurons against cellular degeneration and hypo-trophy. Furthermore, MEME exhibited potent antioxidant activity by dose-dependently reducing MDA levels, but elevating SOD, GSH, and CAT levels (65).

## Therapeutic effects of herb ethanol-extracts on the APDS-induced EPS/EPS-like behaviors and neurobiological changes (**Table 4**)

**Table 4:** Therapeutic effects of ethanol extracts of herbs on EPS/EPS-like behaviors and underlying neurobiological changes.

Herbs	Active ingredients	Subjects	Therapeutic effects	Neurobiological changes	References
Ginkgo biloba (EGb)	Flavonoid glycosides, Terpenoids	SCZ patients, DIP patients; Haloperidol treated rats	TESS subscores 1 & 3↓, Cognitive function↑, VCMs↓	BDNF↑ BAX↓, Bcl-2↑	(18) (66) (16)
Rubia peregrina (ERP)	Tannins, Flavonoids, Glycosides, Reducing Sugar, Steroids, Anthraquinones	Haloperidol treated rats & mice Reserpine treated mice	Catalepsy↓, Orofacial dyskinesia↓	Dopaminergic function ↑	(67) (68)
Korean ginseng (EKG)	Ginsenosides	Reserpine treated rats	VCMs↓, TPs↓, Locomotor activity↑	SOD↑, GSH↑, CAT↑, LPO↓	(71)
Vitex negundo leaves (VNL)	Triterpenes, Diterpenes, Sesquiterpenes, Lignan, Flavonoids, Flavones, Glycosides, Iridoid Glycosides, Stilbene derivative	Haloperidol treated rats	Grip strength↑, Spatial memory & learning↑, Locomotion↑, Cognitive function↑	AChE↓, BChE↓, LPO↓, GSH↑, SOD↑, DA↑	(72)
Plumbago zeylanica L (PZE)	Dopa, Plumbagin, Droseron, Chitranone, Triterpinoid, Anthraquinone	Haloperidol treated rats	Catalepsy score↓, spontaneous ambulatory activity↑	DA ↑, HVA↑	(75) (76)

AChE, acetylcholinesterase; BChE, butyrylcholinesterase; Bcl-2, B-cell lymphoma 2; BDNF,. brain derived neurotrophic factor; CAT, catalase; DA, dopamine; DIP, drug-induced parkinsonism; GSH, glutathione; HVA, homovanillic acid; LPO, lipid peroxidation; SCZ, schizophrenia; SOD, superoxide dismutase; TESS, treatment emergent symptom scale; TPs, tongue protrusions; VCMs, vacuous chewing movements.

### Ethanol extract of *Ginkgo biloba*

EGb is rich in flavonoid glycosides and terpenoids. In a double-blind trial with treatment-resistant schizophrenia patients, co-administration of EGb with haloperidol resulted in markedly reduced behavioral toxicity and neurological symptoms assessed by the Treatment Emergent Symptom Scale (TESS) compared to controls (18). In a randomized controlled clinical trial with drug-induced parkinsonism (DIP) patients, EGb significantly improved cognitive function while elevated brain-derived neurotrophic factor (BDNF) levels (66). In a recent animal study, EGb treatment reduced the haloperidol-induced VCMs, while decreased Bax expression and the Bax/The B-cell lymphoma 2 (Bcl-2) ratio, as well as increased Bcl-2 expression in multiple brain regions of the rat (16).

### Ethanol extract of *Rubia Peregrina*

This ethanol extract contains tannins, flavonoids, glycosides, reducing sugar, steroids, and anthraquinones. In an animal study by Kasture S et al. (67), the *R. peregrina* (100 and 200 mg/kg, i.p.) dose dependently inhibited the haloperidol-induced catalepsy in rats. Similarly, the ethanol extract of *R. peregrina* significantly inhibited haloperidol-induced catalepsy in mice. Moreover, the extract (200 mg/kg, i.p.) significantly inhibited the orofacial dyskinesia induced by intraperitoneal administration of reserpine (1 mg/kg) in mice (68). These therapeutic effects of ERP may be associated with improved dopaminergic neurotransmission.

### Ethanol extract of *Korean ginseng*

*Korean ginseng* (*Panax ginseng*) is the most widely used ginseng, which has the functions of lowering blood pressure, anti-stress, anabolic and cognition enhancing activities (69). Studies have described the beneficial effect of *ginseng* and its main components, ginsenosides, in animal models of PD (70). In an animal study, EKG showed dose-dependent improvements in both motor and memory impairments induced by chronic reserpine administration. Molecular biological analyses revealed its capacity to inhibit lipid peroxidation while preventing the reduction of GSH, SOD, and CAT levels (71).

### Hydroalcoholic extract of *Vitex negundo leaves*

*Vitex negundo Linn* is a member of the Verbenaceae family with antiparkinson activity. The hydroalcoholic extract of leaves *V. negundo* contains triterpenes, diterpenes, sesquiterpenes, lignan, favonoids, favones, glycosides, iridoid glycosides and stilbene derivative (72). The leaves extract shows improving effects on cognitive impairment in rats via inhibiting lipid peroxidation and effecting on AChE in the brain (73, 74). In a recent study, this extract effectively reversed haloperidol-induced behavioral abnormalities in rotarod, water maze, and novel object recognition tests, with particularly pronounced effects being observed at the high oral dose of 400 mg/kg. Histopathological examination revealed its ability to suppress haloperidol-triggered neutrophil infiltration and nuclear fragmentation while restoring neuronal structural integrity in the corticolimbic system. Further investigations have shown that this extract has a remarkable ability to restore GSH, SOD and DA levels, while reducing the content of lipid peroxidation (LPO) and the activities of AChE and BChE, indicating its crucial role in regulating the pathophysiology of EPS (72).

### Hydroalcoholic extract of *plumbago zeylanica leaves*

With the bioactive components of L-dopa, plumbagin, droseron, chitranone, triterpinoid, and anthraquinone, the hydroalcoholic extract of PZL effectively reduced haloperidol-induced elevation of catalepsy in Wistar rats (75). In another animal study, the extract of the PZL specifically enhanced the spontaneous ambulatory activity without inducing stereotypic behavior, while elevated levels of DA and HVA in striatum of rats compared with the controls (76).

## Therapeutic effects of aqueous extract of individual herbs on the APDs-induced EPS-like behaviors and neurobiological changes (**Table 5**)

**Table 5:** Therapeutic effects of aqueous extracts of herbs on EPS-like behaviors and the underlying neurobiological changes. .

Herbs	Active ingredients	Subjects	Therapeutic effects	Neurobiological changes	References
Nardostachys jatamansi (N. jatamansi)	Sterols, Carbohydrates, Terpenes, Phenols, Gums, Mucilage	Haloperidol treated rats	Catalepsy score↓	TBARS↓, SOD↑, CAT↑, GSH↑	(27)
Sea buckthorn (SBT)	Vitamin C, Carotene, Flavonoids, Phytosterols, Essential amino acids	Haloperidol treated rats	Exploratory activity↑, Catalepsy score↓, VCMs↓	DA↑, DOPAC↓, HVA↓	(79)
Bauhinia forficata (B. forficata)	Gallic acid, Catechin, Caffeic acid, Rosmarinic acid, Rutin, Isoquercitrin, Quercitrin, Quercetin, Kaempferol	Haloperidol treated rats	VCMs↓, Crossings↓, Rearings↓	TBARS↓	(83)
Withania somnifera	Withanolides	Haloperidol treated rats, Reserpine treated rats	Catalepsy score↓, VCMs↓, TPs↓, Memory↑	SOD↑, MDA↓, GSH↑, CAT↑, SOD↑	(84) (85)
Ilex paraguariensis	Methylxanthines, Triterpene saponins, Polyphenols, Flavonoids, Vitamins	Haloperidol treated rats,	VCMs↓, Spatial memory function↑	TBARS↓	(28)

CAT, catalase; DA, dopamine; DOPAC, dihydroxyphenylacetic acid; HVA, GSH, glutathione; MDA, malondialdehyde; SOD, superoxide dismutase; TBARS, thiobarbituric acid reactive substances; TPs, tongue protrusions; VCMs, vacuous chewing movements.

### Aqueous extract of roots of Nardostachys jatamansi

This extract of *N. jatamansi* contains sterols, carbohydrates, terpenes, phenols, gums and mucilage (27). It exhibits potent antioxidant activity and has been widely used in treating neurological disorders such as depression (77) and convulsions (78), as well as cardiovascular diseases. In the haloperidol-induced catalepsy rat model, oral administration of this extract significantly reversed the haloperidol-induced catalepsy. The maximal decrease in catalepsy was observed in the group receiving the extract at a dose of 250 mg/kg. In addition, the treatment significantly restored the peroxides and antioxidant levels (GSH, SOD, and CAT levels) to near normal levels in the brains of the haloperidol-exposed rats (27).

### Aqueous extract of *sea buckthorn*

*Sea buckthorn* (*Hippophae rhamnoides*) offers many health benefits. The aqueous extract of it contains vitamin C, carotene, flavonoids, phytosterols, and all essential amino acids. In a rat model of TD, *hippophae rhamnoides* fruit extract showed a protective role against haloperidol-induced orofacial dyskinesia. Mechanistically, the treatment increased striatal DA levels while suppressing its metabolic degradation, as evidenced by reduced concentrations of the DA metabolites HVA and 3, 4-dihydroxyphenylacetic acid (DOPAC) (79). In an *in vitro* study, *sea buckthorn* leaf extracts protected neuronal PC-12 cells from oxidative stress (80). Related to this mechanism, polysaccharides from *sea buckthorn* berries ameliorated cognitive dysfunction in AD mice by suppressing oxidative stress and inflammation reaction (81).

### Aqueous extract of *Bauhinia forficate*

*Bauhinia forficata* is a plant rich in polyphenols that has been used mainly for its hypoglycemic activity, which is related to its antioxidant and anti-inflammatory potential (82). In an animal model of orofacial dyskinesia induced by long-term treatment with haloperidol (38 mg/kg for consecutive 28 days), co-treatment with *B. forficata* partially prevented the haloperidol-induced VCMs, while decreasing thiobarbituric acid reactive substances (TBARS) levels, suggesting its potential utility in treating EPS (83).

### Aqueous extract of *Withania somnifera*

This extract contains substantial amounts of withanolides. In *albino* mice treated with haloperidol, this extract showed a dose dependent reduction in catalepsy scores, and this effect was more efficacious than scopolamine. And a clear correlation between the SOD levels and catalepsy scores was observed. The authors believed that the antioxidant properties of the aqueous extract of *W. somnifera* could have contributed to the anticataleptic effect (84). In another animal study, chronic treatment with this extract for 4 weeks to reserpine treated rats significantly and dose dependently (50 and 100 mg/kg) reduced the reserpine-induced VCMs and TPs. Moreover, chronic administration of *W. somnifera* significantly reversed reserpine-induced retention deficits. Biochemical analysis revealed that chronic administration of *W. somnifera* root extract dose dependently (50 and 100 mg/kg) reduced the lipid peroxidation and restored the decreased GSH levels by chronic reserpine treatment. It also significantly reversed the reserpine-induced decrease in brain SOD and CAT levels in rats (85).

### Aqueous extract of *Ilex paraguariensis*

This extract possesses well-documented diuretic, anti-inflammatory and antioxidant properties that contribute to its therapeutic applications for various medical conditions (86). It contains substantial quantities of compounds. In a rat model receiving weekly haloperidol administration (12 mg/kg) for 4 weeks, the extract significantly attenuated the haloperidol-induced VCMs and ameliorated memory impairments detected in water-maze tests, while reducing TBARS levels. These findings suggest its potential utility in managing EPS related movement disorders through antioxidant mechanism (28).

## Therapeutic effects of flavonoids on the APDs-induced EPS-like behaviors and neurobiological changes (**Table 6**)

**Table 6:** Therapeutic effects of flavonoids on EPS-like behaviors and the underlying neurobiological mechanisms.

Classic flavonoids	Subjects	Therapeutic effects	Neurobiological changes	References
Vitexin (VTX)	Haloperidol treated rats	VCMs↓, TPs↓	Nitrite↓, TBARS↓, GSH↑, SOD↑, CAT↑, SDH↑, Total ATPase↑, Complexes I-III, Complexes II-III↑, TNF-α↓, IL-1β↓, IL-6↓, Caspase-3↓	(95)
Baicalin	MPTP treated mice Caenorhabditis elegans	Performance in rotarod and grid tests↑, Reversals↑, Omega turns↑	DA neurons ↑, OS↓ Proinflammatory cytokines ↓ MDA↓ CAT↑, GR↑, GSH↑, SOD↑	(96) (97)
Baicalein	MPTP-treated mice	Spontaneous motor activity ↑, Performance in the pole & rotarod tests↑	levels of DA and 5-HT↑ DA neuron ↑, OS, and astroglia response ↓ TNF-α and IL-1β ↓ *limk1*, *snca* & *glra1*↑	(98) (99) (100) (101)
Quercetin	Haloperidol treated rats Perphenazine or reserpine treated rats	VCMs↓, TPs↓, Catalepsy duration↓	MDA↓, GSH↑, SOD↑, CAT↑, MAO↓, COMT↓	(22) (23)
Rutin	Haloperidol treated rats 3-NP treated rats Haloperidol-treated rats	VCMs↓, TPs↓, FJs↓, Locomotor activity↑, Spatial long term memory↑, Muscle coordination↑, Catalepsy duration↓	Improved oxidative damage DA↓, SOD↑, CAT↑, GSH↑, GPx↑, GST↑, GR↑, AChE↓, DA↑, NE↑, Oxidative damage ↓	(102) (103) (104)
Icariin	Haloperidol treated rats MK-801 treated mice	Spontaneous locomotor activity↑ Anxiety↓, Recognition memory↑, Motor coordination↑, Prepulse inhibition↑, Social interaction↑	GSK-3β↓, MDA↓, GST↑, CAT↑, SOD↑, NE↑, DA↑, TH↑, 5-HT↑, Grey matter atrophy↓, Cell apoptosis↓, ATP1B2↑, p-PI3K/PI3K↑, p-Akt/Akt↑, p-mTOR/Mtor↑	(105) (106) (107)
Hesperetin	Haloperidol treated rats Ketamine treated mice	VCMs↓, TPs↓, Catalepsy score↓, Locomotor activity↓, Schizophrenia-like behaviors↓	DA↑, 5-HT↑, SOD↑, GSH↑, CAT↑, TBARS↓	(26) (108)
Naringin	Haloperidol treated rats	VCMs↓, TPs↓	Nitrite↓, TBARS↓, SOD↑, GSH↑, CAT↑, TNF-α↓, IL-1β↓, IL-6↓, Caspase-3↓, DA↑, DOPAC↓, HVA↓, NE↑, 5-HT↑, 5-HIAA↑	(25)
Isoflavones Chalcones	Haloperidol treated rats Haloperidol treated mice	Hypokinesia↓, VCMs↓, Extrapyramidal and non-motor symptoms↓, Locomotory and emotor coordination↑	IL-1β↓, TNF-α↓, SOD↑, GSH↑, CAT↑, MDA↓, Nitrite↓, DA↑, NA↑, 5-HT↑, AChE↑, NT↓, Plaques↓, MAO-B↓	(109) (110) (112)

5-HIAA, 5-hydroxyindole-3-acetic acid; 5-HT, serotonin; AChE, acetylcholinesterase; Akt, protein kinase B; ATP1B2, Beta2-subunit of Na(+)/K(+)-ATPase; CAT, catalase; COMT, catechol-O-methyltransferase; DA, dopamine; GPx, glutathione peroxidase; GR, glutathione reductase; GSH, glutathione; GST, glutathione-S-transferase; HVA, homovanillic acid; IL-1β, interleukin-1β; IL-6, interleukin-6; MAO-B, monoamine oxidase B; MDA, malondialdehyde; NA, noradrenaline; NE, norepinephrine; NT, neurofibrillary tangle; p-Akt, phosphorylated protein kinase B; PI3K, phosphatidylinositol 3 kinase; p-PI3K, phosphorylated phosphatidylinositol 3 kinase; SOD, superoxide dismutase; TBARS, thiobarbituric acid reactive substances; TNF-α, tumor necrosis factor-α; TPs, tongue protrusions; VCMs, vacuous chewing movements.

Flavonoids represent a class of naturally occurring polyphenolic compounds ubiquitously distributed in plants, characterized by their fundamental C6-C3-C6 skeleton comprising two benzene rings connected by a three-carbon chain. These compounds have been extensively investigated for their diverse pharmacological properties including anti-inflammatory, antioxidant, antimicrobial, anticancer, and cardiovascular activities (87–89). In EPS research, flavonoids are systematically classified into classical flavonoids, flavonols, dihydroflavonoids, isoflavones, and chalcones based on three key structural features: the oxidation state of the central three-carbon chain, the attachment position of the B-ring (C2 or C3), and the cyclization status of the central chain.

### Classical flavonoids

This kind of flavonoids are structurally characterized by the 2-phenylchromone skeleton (B-ring attached at C2 position), C2-C3 double bond, and C4 carbonyl group, exhibiting primary bioactivities including anti-inflammatory, antioxidant, and antitumor effects. Of the classical flavonoids, here we introduced vitexin, baicalin and baicalein.

Vitexin is a C-glycosylated flavone found in various medicinal herbs and known for its antioxidant, anti-inflammatory, and neuroprotective properties. The antioxidant properties of vitexin contribute to its neuroprotective effects against various behavioral impairments, including seizures, depression-like symptoms, and motor and memory dysfunctions caused by ischemia, toxins, and stress (90–94). In a recent animal study, vitexin ameliorated haloperidol-induced orofacial dyskinesia in rats while alleviated neuroinflammation, nitrosative and oxidative damage, and mitochondrial dysfunction, potentially mediated through the nuclear factor erythroid 2-related factor 2 (Nrf2) pathway in rats (95).

Baicalein and baicalin are the principal active flavonoid components derived from *Scutellaria baicalensis* (*S. baicalensis*). In a 1-methyl-4-phenyl-1, 2, 3, 6-tetrahydropyridine (MPTP) induced PD mouse model, baicalin improved the PD model’s behavioral performance in rotarod test and grid test and reduced dopaminergic neuron loss in the substantia nigra, associated with the inactivation of proinflammatory cytokines and oxidative stress (96). In a *Caenorhabditis elegans* model of PD, baicalin improved the reversal and omega turn behavioral phenotypes, as well as the survival of 6-OHDA-stimulated worms. It also inhibited 6-OHDA-induced oxidative stress by decreasing MDA levels, increasing levels of CAT, SOD, glutathione reductase (GR), and GSH and up-regulating mRNA levels of the antioxidant-related genes *sod-1, sod-2, sod-3, daf-2*, and *daf-16* (97).

In an animal study, baicalein improved the spontaneous motor activity decrease and a marked prolongation of latent period in the pole test in MPTP-treated mice, while increased the levels of DA and 5-HT in the striatum, alleviated the dopaminergic neuron decrease, oxidative stress, and astroglia response in brains of the mice (98). In another animal study, baicalein improved the MPTP-induced impairment in spontaneous motor activity and rotarod performance, while exhibited a protective effect against the MPTP-induced decreases in TH-positive fibers in the substantia nigra and DA levels (99). Moreover, baicalein significantly improved the behavioral performance of MPTP-treated mice in the rotarod task while attenuated the upregulation of striatal basal glutamatergic strength and decreased the upregulation of cytokines (TNF-α and IL-1β) in the substantia nigra and striatum in the mice (100). Similarly, baicalein significantly improved the abnormal behaviors in MPTP-induced mice model of PD, as manifested by shortening the total time for climbing down the pole, prolonging the latent periods of rotarod, and increasing the vertical movements. In the meanwhile, baicalein significantly regulated the expression of genes such as LIM-kinase 1 (LIMK1), alpha-synuclein gene (SNCA) and glycine receptor, alpha 1 subunit (GLRA1) (101).

### Flavonols

Of the flavonols, here we introduce quercetin, rutin, and icariin.

Quercetin exhibits potent antioxidant properties. In a study by Pattipati et al., quercetin dose-dependently reversed haloperidol-induced lipid peroxidation, restored GSH, SOD, and CAT activity in the rat brain, and alleviated haloperidol-induced VCMs and TPs in rats (22). In another animal study, either 5 mg/kg/day of perphenazine or 2.5 mg/kg/day of reserpine significantly increased muscle catalepsy in rats, as evidenced by prolonged immobility time in the catalepsy bar test. Co-administration of quercetin markedly alleviated these abnormal behaviors, which were hypothesized to be linked to the inhibition of MAO and catechol-O-methyltransferase (COMT) activity (23).

Rutin is another well-known flavonoid from citrus fruits. In an earlier animal study, rutin significantly inhibited haloperidol-induced VCMs, TPs and FJs in rats. Additionally, rutin prevented the haloperidol-exposed rats from oxidative damage in all regions of the brain, especially in the striatum (102). In the 3-nitropropionic acid (3-NP) induced experimental model of HD, 3-NP significantly reduced locomotor activities, memory and antioxidants including GSH, SOD, CAT, GPx, GST, and GR. Rutin treatment (25 and 50 mg/kg) significantly restored all the biochemical and behavioral changes caused by the 3-NP through its antioxidant activity (103). In another animal study, rats treated with the rutin nanoemulsion exhibited significantly greater activity, better muscle coordination and improvement in cataleptic behaviour compared to the normal and haloperidol-induced rats. Rutin nanoemulsion also ameliorated oxidative stress damage as indicated by increased levels of GSH, SOD and decreased MDA in the brain (104).

Icariin is a flavonoid derived from *Herba Epimedii*. In a recent study, icariin attenuated dopaminergic neuron loss in haloperidol-induced Parkinsonism in rats via upregulating TH and restoring monoaminergic neuron number in the cortex and midbrain, thereby ameliorating haloperidol-induced pathological behavioral manifestations such as reduced spontaneous locomotor activity (105). Significantly, icariin alleviated MK-801-induced anxiety and recognition memory deficits in rats. Additionally, weakened motor coordination caused by MK-801 was restored by icariin. Furthermore, brain grey matter atrophy, cytotoxicity, and cell apoptosis caused by MK-801 were eliminated by icariin. Lastly, icariin regulated the expression of miR-144-3p and ATP1B2, and enhanced the phosphorylation of PI3K, Akt, and mTOR (106). Importantly, icariin and its two metabolites, successfully mitigated MK-801-induced schizophrenia-like symptoms, including deficits in prepulse inhibition and social interaction, in mice (107).

### Dihydroflavones

Dihydroflavones are formed by the partial hydrogenation of the C2-C3 single bond in flavonols and are primarily derived from citrus fruit peels, being utilized for their antioxidant and antifibrotic properties. Currently, hesperidin (hesperetin) and naringin are among the compounds being studied for their potential in treating EPS.

Hesperetin is mostly present in citrus fruits and has a number of pharmacological actions, such as those against liver fibrosis, cancer, and hyperglycemia. In a haloperidol-induced rat model of EPS, hesperetin significantly reversed haloperidol- induced orofacial dyskinesia and this protective effect was comparable to that of quercetin. In addition, hesperetin administered for 21 consecutive days significantly ameliorated haloperidol-induced oxidative stress, as indicated by increased activities of brain SOD, GSH, and CAT, along with a decrease in brain MDA levels. These findings suggest hesperetin is a potential therapeutic agent for clinically managing neuroleptic-induced TD (26). Interestingly, nano-hesperetin showed a stronger neuroprotective impact than hesperetin. In cerebral cortex tissues, nano-hesperetin treatment dramatically reduced ketamine-induced schizophrenia-like behavior and oxidative stress indicators (108).

Naringin is a bioflavonoid commonly found in citrus fruits and has potent antioxidative, anti-inflammatory, anti-apoptotic, and neuroprotective properties. In a rat model of human TD, naringin (100 and 300 mg/kg) prevented haloperidol-induced orofacial dyskinesia significantly. Moreover, naringin treatment reduced the haloperidol-induced nitric oxide and lipid peroxide production, increased the antioxidation power and neurotransmitter levels in the striatum, and significantly reduced the levels of neuroinflammatory and apoptotic markers. These findings suggest that naringin may help delay or treat human TD in clinical settings (25).

### Isoflavones

Isoflavones are a class of natural polyphenol compounds primarily found in legumes (especially soybeans). Due to their chemical structure being similar to human estrogen, they are often referred to as “phytoestrogens.” The most common isoflavones in soybeans include genistein, daidzein, and glycitein. Different from classical flavonoids, Isoflavones have their B-ring attached at the C3 position rather than C2. In haloperidol-treated rats, soy isoflavones reversed hypokinesia and VCMs while reducing elevated levels of pro-inflammatory factors IL-1β and TNF-α (109).

### Chalcones

Chalcones are also termed open-chain flavonoids due to their unclosed C-ring, a single bond between C2-C3. In a haloperidol-induced murine model of PD, the compounds O10 and O23 (two of synthesized 26 chalcone compounds) ameliorated extrapyramidal and non-motor symptoms, while reduced oxidative stress markers, and enhanced antioxidant marker and neurotransmitter levels (110). In a recent comprehensive review, chalcone was associated with multiple functions, including inhibitors of MAO-B, COMT, and AChE, alpha synuclein imaging probes, inhibitors of inducible nitric oxide synthase (iNOS) or Nrf2 signaling, and antagonists of adenosine A1 and/or A2A receptors (111). In addition, the ethoxylated chalcone derivative (E)-1-(4-ethoxyphenyl)-3-(fluorophenyl) prop-2-en-1-one (E7) has potent, reversible, and competitive MAO-B inhibition. In haloperidol-exposed mice, the E7 compound exhibited anti-Parkinson activity as evidenced by a marked improvement in locomotor activity and motor coordination in the animals (112).

## Therapeutic effects of phenolic compounds on the APDs-induced EPS/EPS-like behaviors and neurobiological changes (**Table 7**)

**Table 7:** Therapeutic effects of phenolic compounds on EPS/EPS-like behaviors and the underlying neurobiological changes.

Phenolic compounds	Subjects	Therapeutic effects	Neurobiological changes	References
Curcumin	Haloperidol treated rats	VCMs↓, TPs↓, FJs↓, Stereotypic behaviors↓,	Oxidative damage↓ DA↑, 5-HT↑, NE ↑	(19) (20)
Resveratrol	6-OHDA treated rats, Reserpine treated mice, Methamphetamine treated mice, Fluphenazine treated rats, Transgenic drosophila melanogaster, A53T transgenic mice	Rotations↓, Coordination↑, Steps↑, VCMs↓, Locomotor and exploratory activity↑, Hyperactivity↓, Lifespan↑, locomotor activity↑	TBARS↓, GSH↑, SOD↑, CAT↑, GPx↑, GR↑, DA↑, DOPAC↑, MAO-A↑, MAO-B↑, AChE↑, NO↓, MDA↓, Sod1↑, Mitochondrial function↑, VDAC1↓, α-syn↓, ROS↓,	(113) (114) (115) (116) (117) (118)
Cannabidiol (CBD)	Haloperidol treated Zebrafish, Haloperidol treated rats	Distance traveled↑, VCMs↓, Locomotor activity↑, Catalepsy↓	DA neurons↑, Inflammation↓, Microglial/astrocyte activation↓, OS↓	(121) (122) (123)
Epicatechin	Healthy human peripheral plasma treated with haloperidol; Haloperidol treated mice	/ Catalepsy↓, Locomotor activity↑	TBARS↓, Lipid peroxide↓	(124) (125)
Aronia berry polyphenols	Ziprasidone-treated patients	/	Plasma TBARS↓	(124)

5-HT, serotonin; AChE, acetylcholinesterase; α-syn, alpha-Synuclein; CAT, catalase; DA, dopamine; DOPAC, 3, 4-Dihydroxyphenylacetic acid; FJs, facial jerkings; GPx, glutathione peroxidase; GR, glutathione reductase; GSH, glutathione; MAO-A, monoamine oxidase A; MAO-B, monoamine oxidase B; MDA, malondialdehyde; NE, norepinephrine; NO, nitric oxide; OS, oxidative stress; ROS, reactive oxygen species; SOD, superoxide mutase; Sod1, superoxide dismutase 1 gene; TBARS, thiobarbituric acid reactive substances; TPs, tongue protrusions; VCMs, vacuous chewing movements; VDAC1, voltage-dependent anion channel 1.

Phenolic compounds are a class of substances composed of one or more aromatic rings bonded with one or more hydroxyl groups. The hydroxyl groups on their benzene rings readily lose hydrogen electrons, making them excellent electron donors that exert antioxidant functions. Research has been done to explore possible applications of these phenolic compounds in treating EPS/EPS-like behaviors.

### Curcumin

Derived from turmeric, curcumin is a polyphenolic compound that modulates multiple cell signaling pathways. Extensive clinical trials over the past decades have addressed the pharmacokinetics, safety, and efficacy of this nutraceutical against numerous diseases in humans. In rats exposed to haloperidol for 21 consecutive days, the EPS phenotypes of VCMs, TPs, FJs, and stereotypic behaviors were effectively alleviated by curcumin. In addition, curcumin reversed haloperidol-induced oxidative stress and restored DA, 5-HT, and NE turnover, particularly in subcortical regions including the striatum (19). Moreover, combination of curcumin (25 mg/kg) and piperine (2.5 mg/kg) not only improved oxidative stress but also reduced inflammatory and apoptotic markers such as TNF-α, NF-κB p65 levels, and caspase activity (20).

### Resveratrol

As a polyphenolic compound, resveratrol is a sirtulin activator that has been shown to regulate dopaminergic systems thereby contributing to the behavioral effects of methamphetamine and cocaine. In earlier animal studies, resveratrol attenuated 6-OHDA-induced oxidative damage and DA depletion in a rat model of PD (113). Resveratrol protected against a model of VCMs induced by reserpine in mice (114). Repeated resveratrol treatment (1–20 mg/kg) decreased methamphetamine (0.5 mg/kg)-induced hyperactivity in mice. The treatment also attenuated the methamphetamine’s (0.1-60 μM) efficacy to evoke ^3^H overflow from rat striatal slices preloaded with ^3^H-DA (115). In addition, resveratrol protected against VCMs induced by chronic treatment with fluphenazine (116). In a transgenic drosophila melanogaster model expressing human α-synuclein, resveratrol supplementation in the diet significantly improved lifespan, locomotor activity, AChE and CAT activities, and thiol content compared to untreated PD flies. Furthermore, resveratrol reduced nitric oxide (nitrite/nitrate), MDA, and total hydroperoxide levels, and enhanced cellular metabolic activity and upregulated *Sod1* mRNA expression (117). Of note, resveratrol inhibits voltage-dependent anion channel I (VDAC1)-mediated mitochondrial dysfunction to mitigate pathological progression in PD model (118). Aligning with these *in vivo* studies, resveratrol prevented haloperidol-induced mitochondria dysfunction by inducing autophagy in SH-SY5Y cells (119).

### Cannabidiol

It is a cannabis constituent and has a pharmacological profile similar to that of atypical APDs (120). Interestingly, at concentrations equivalent to half of haloperidol’s, CBD almost completely reversed haloperidol-induced motor dysfunction in zebrafish via D2 receptor blockade (121). In a rat model of TD, oral CBD (5 mg/kg) attenuated the VCMs produced by sub-chronic administration of haloperidol (5 mg/kg) but had minimal effects on the VCMs produced by chronic administration of haloperidol (50 mg/kg) (122). Furthermore, the use of phytocannabinoids improved locomotor activity and involuntary movement and reduced catalepsy in animal studies. In addition, there was an improvement in the evaluation of dopaminergic neurons. And inflammation, microglial/astrocyte activation and oxidative stress were reduced after treatment with phytocannabinoids (123).

### Epicatechin

It is a major component of green tea. In a previous human study, epicatechin significantly reduced haloperidol-elevated TBARS in human peripheral plasma, outperforming both quercetin and resveratrol (124). Related to this is, administration of epigallocatechin gallate (EGCG) inhibited haloperidol-induced catalepsy, and a combination of caffeine and EGCG produced greater inhibition of haloperidol-induced catalepsy in mice. Furthermore, EGCG administration reduced striatal lipid peroxide levels in a dose-dependent manner (125).

## Therapeutic effects of alkaloids on the APDs-induced EPS/EPS-like behaviors and neurobiological changes (**Table 8**)

**Table 8:** Therapeutic effects of alkaloids on EPS/EPS-like behaviors and the underlying neurobiological changes.

Alkaloids	Subjects	Therapeutic effects	Neurobiological changes	ReferencesA
Betaine	Haloperidol treated rats	VCMs↓, TPs↓	TBARS↓, Nitrite↓, SOD↑, GSH↑, CAT↑, SDH↑, total ATPase↑, Complexes I-III ↑, TNF-α↓, IL-1β↓, IL-6↓, Caspase-3↓	(24)
Berberine	3-NP treated rats or haloperidol, Rotenone treated rats	Motor coordination↑, locomotor activity↑, VCMs↓, TPs↓, FJs↓, Motor deficits↓, Weight reduction↓	MDA↓, SOD↑, GSH↑, CAT↑, Nitrite↓, DA↑, MAO activity↓	(131) (132)
Murrayanine (MK)	Haloperidol treated rats	VCMs↓, TPs↓, FJs↓	SOD↑, CAT↑, LPO↓, GSH↑	(133)
Caffeine Theophylline	Reserpine treated mice, Reserpine treated rats, Haloperidol treated mice Haloperidol treated C. apella monkeys	Akinesia↓, Locomotion↑, Exploratory activity↑, Catalepsy↓, Swim ability↑	DA↑, GSH↑, SOD↑	(135) (136) (137) (138)
	Parkinsonian patients, ADCY5-related dyskinesia patients; Haloperidol treated rats	GCRS↓, UPDRS↓, Life quality↑, Movement disorder↓; VCMs↓, TPs↓, FJs↓, Stereotypy↓, Locomotor activity↑	/ / LPO↓, GSH↑, CAT↑, SOD↑	(139) (140) (141)
Piperine	MPTP treated mice 6-OHDA treated rats 3-NP treated rats	Motor coordination↑, Cognitive functioning↑, Motor coordination and balance↑	TH^+^ cells↑, Microglia activation↓, IL-1β↓, LPO↓, GSH↑, MAO↑, 5-HT↑, GFAP↓	(144) (145) (146) (147)

3-NP, 3-nitropropionic acid; 5-HT, serotonin; 6-OHDA, 6-hydroxydopamine; ADCY5, adenylyl cyclase 5; CAT, catalase; DA, dopamine; FJs, facial jerkings; GFAP, glial fibrillary acidic protein; GCRS, glucose control resistance scale; GSH, glutathione; IL-1β, interleukin-1β; IL-6, interleukin-6; LPO, lipid peroxidation; MAO, monoamine oxidase; MDA, malondialdehyde; MPTP, 1-methyl-4-phenyl-1, 2, 3, 6-tetrahydropyridine; SDH, succinate dehydrogenase; SOD, superoxide, dismutase; TH, tyrosine hydroxylase; TNF-α, tumor necrosis factor-α; TPs, tongue protrusions; UPDRS, unified Parkinson’s disease rating scale; VCMs, vacuous chewing movements.

Alkaloids are nitrogen-containing basic organic compounds widely found in nature, most of which possess complex cyclic structures and exhibit significant biological activity. They represent one of the important active components in phytomedicines.

### Betaine

BT is also known as glycine betaine found from Beta vulgaris (beet) that is abundantly present in various food sources like wheat bran, spinach, and seafood. It exhibits various pharmacological effects, including antioxidant and anti-inflammatory activities, as well as the ability to mitigate endoplasmic reticulum stress and apoptosis (126). Earlier animal studies reported that betaine prevents ethanol-induced oxidative stress and reduces total homocysteine in the rat cerebellum (127) and protects cerebellum from oxidative stress following levodopa and benserazide administration in rats (128). In a recent animal study, BT was found to prevent the haloperidol-induced orofacial dyskinesia, decrease oxidative stress levels, increase anti-oxidation power, prevent mitochondrial dysfunction, and reduce the levels of neuroinflammatory and apoptotic markers in the striatum of rats (24).

### Berberine

Berberine is an isoquinoline alkaloid derived from several medicinal plants, including *Berberis aristata* and *Coptis chinensis*. It has been shown to enhance the endogenous antioxidant enzymes such as CAT, GPx, and SOD, which are critical in neutralizing ROS and preventing cellular damage (129). In addition, berberine was shown to inhibit ROS overproduction in an *in vitro* system, contributing to its role as a powerful antioxidant (130). In a recent animal study, berberine attenuated 3-NP and haloperidol-induced TD and improved the antioxidant capacity in rats (131). Moreover, combined administration of berberine (25 mg/kg/day) and caffeine (2.5 mg/kg/day) for four weeks prevented motor deficits, weight reduction, and DA depletion in rats exposed to rotenone compared to monotherapy of berberine or caffeine in a more recent study (132).

### Murrayanine

Murrayanine is the main compound isolated from *Murraya koenigii* leaves, an aromatic plant belonging to the *rutaceae* family. Murrayanine was reported to possess potential antioxidant, antimycobacterial and antifungal effects. In an earlier animal study, rats exposed to 1 mg/kg haloperidol for 21 days developed hypolocomotion and orofacial dyskinesia (VCMs, TPs, FJs), accompanied by increased lipid peroxidation and decreased forebrain GSH, SOD, and CAT levels. Co-administration of *M. koenigii* leaves and its alkaloid fraction reversed the motor deficits, restored antioxidant enzyme levels, and prevented LPO accumulation, highlighting its therapeutic potential for EPS (133).

### Caffeine and theophylline

Caffeine and theophylline are primary components of green tea. They share very similar chemical structures and pharmacological effects of strong antioxidant activity. Meta-studies have shown that regularly drinking caffeine or caffeinated coffee significantly reduces the risk of developing AD, epilepsy, and (134). In support of the observations in humans, the reserpine-induced akinesia in mice was significantly and dose-dependently reversed by the methylxanthine and caffeine. This anti-akinetic effect of caffeine within a pattern of catecholamine depletion has been interpreted as a DA mimetic activity (135). In rats, treatment with subthreshold doses of caffeine plus trihexyphenidyl fully restored locomotion and exploratory activity in reserpinized rats (136). In a preclinical model of PD, caffeine pre-treatment significantly decreased haloperidol-induced catalepsy, akinesia and swim disability in mice (137). In primates, caffeine (10 mg/kg) significantly reduced haloperidol-induced catalepsy (138).

Similar to caffeine, the effect of theophylline on parkinsonian symptoms has been reported in humans (139). In a recent retrospective study with 12 patients with ADCY5-related dyskinesia (a rare neurological disease caused by mutations in the gene encoding the adenylyl cyclase 5 (ADCY5) isoform, theophylline improved various aspects of patients’ quality of life and movement disorder symptoms, showing substantial promise as a treatment option for ADCY5-related dyskinesia (140). In an animal model of TD, theophylline prevented behavioral (orofacial dyskinetic movements, stereotypy, locomotor activity), biochemical (LPO, GSH, CAT and SOD), and neurotransmitter (DA) changes in rats (141).

### Piperine

Piperine is an alkaloid derived from “*Piper nigrum*”, the plant source of both black and white peppercorns. Pharmacological studies have reported that piperine possesses anticancer and antioxidative properties (142, 143). Piperine has also been reported to inhibit toxicity of 1-methyl-4-phenylpyridinium (MPP+)-induced mitochondrial dysfunction and cell death in PC12 cells (144). Via the antioxidative property, piperine treatment improved the MPTP-induced deficits in motor coordination and cognitive functioning in mice, while prevented MPTP-induced decreases in the number of tyrosine hydroxylase-positive cells in the substantia nigra of the animals. Moreover, piperine inhibited microglia activation and decreased levels of IL-1β, and oxidative stress following MPTP treatment (145). Moreover, piperine reduced 6-OHDA-induced LPO and stimulated glutathione levels in striatum, while improved motor coordination and balance behavior in rats (146). And piperine mitigated behavioral impairments and exerted neuroprotection against 3-NP-induced HD-like symptoms (147).

## Therapeutic effects of terpenoids on the APDs-induced EPS-like behaviors and neurobiological changes (**Table 9**)

**Table 9:** Therapeutic effects of terpenoids on EPS-like behaviors and the underlying neurobiological changes. .

Terpenoids	Subjects	Therapeutic effects	Neurobiological changes	References
Myrtenol	Reserpine treated mice	Catalepsy↓, VCMs↓, Short-term memory↑	TBARS↓, TAS↑, TOS↓, TH↑	(151)
Menthol	LPS treated mice	Ambulation ↑ Rotational behavior↓	/ Alleviated DA neuron loss & TH decrease Neuroniflammation↓	(152) (153) (154)
Carvacrol	6-OHDA treated mice 6-OHDA treated rats Reserpine treated rats	Forelimbs asymmetrical use↓, Catalepsy↓, Akinesia↓, Motor coordination↑, Catalepsy↓, VCM↓	TH↑, Caspase-3 level↓ GSH↑, MDA↓, SOD↑, TH immunostaining↑	(155) (156) (157)
Glycyrrhizic Acid (GA)	MPTP treated mice Haloperidol treated rats MPTP treated mice	/ VCMs↓, TPs↓, FJs↓ Improved motor function & alleviated gait disturbances	HMGB1↓, RAGE↓ GSH↑, SOD↑, MDA↓, Attenuated dopaminergic neuronal degeneration	(158) (159) (160)
Oleanolic acid (OA)	6-OHDA treated rats Haloperidol-treated mice Rotenone treated mice Mice treated with MPTP	Forelimb use asymmetry↓ Balance function↑, Catalepsy↓, VCMs↓, Cognitive function↑ Improved motor and non-motor function Prevented PD-like motor symptoms	DA↑ Normalized DA & Ach levels in the stratum IL-1β, TNF-α, IL-6, & OS↓ NE, DA, & 5-HT↑ Neuronal death ↓ Bcl-2 and DJ-1 levels↑	(161) (162) (163) (164)
Lycopene	MPTP-treated mice 6-OHDA-treated rats Rotenone-treated mice MPTP-treated mice Haloperidol-treated rats	/ / Reversed cognitive & motor deficits Motor impairments ↓ VCMs↓, TPs↓, FJs↓, Muscle coordination↑	Prevented DA decrease in the striatum Levels of DA, DOPAC, HVA in the striatum↑ Activities of SOD, GPx & CAT↑ DA↑, DOPAC↑ HVA↑ DA↑, 5-HT↑, NE↑, 5-HIAA↑ TNF-α↓, IL-1β↓, IL-6↓	(165) (166) (167) (168) (169)
Crocin	Haloperidol treated rats Malathion treated rats Rotenone-treated rats Cisplatin-treated rats Haloperidol-treated rats MPTP-treated mice Rotenone-treated mice	VCMs↓, TPs↓, Locomotor and exploratory activity↑ Cataleptic immobilization ↑, Locomotor activity↑, Muscular coordination↑ Improved performance in bar and grid tests, Postural instability↑ Avoidance memory↑, Explorative behavior ↑, Motor coordination ↑ Catalepsy↓, Orofacial dyskinesia↓ Performance in rod test, pole test, and hanging test ↑ Anxiety ↓, exploratory behaviour ↑, motor co-ordination ↑	MDA↓, GSH↑ AChE ↑, GSH↑, Lipid peroxidation↓, IL-6↓, TNFα ↓ DA↑, TH ↑, α-synuclein ↓ CAT↑, GPx↑, MDA↓ GSH ↑, MDA↓, TNFα ↓, IL1β ↓ TH-positive neurons↑ ROS ↓, hydroperoxides↓, MDA ↓	(173) (174) (175) (176) (177) (178) (179)

5-HT, serotonin; 6-OHDA, 6-hydroxydopamine; AChE, acetylcholinesterase; ADCY5, adenylyl cyclase 5; CAT, catalase; DA, dopamine; DOPAC, 3, 4-Dihydroxyphenylacetic acid; FJs, facial jerkings; GFAP, glial fibrillary acidic protein; GCRS, glucose control resistance scale; GPx, glutathione peroxidase; GSH, glutathione; HIAA, 5-hydroxyindoleacetic acid; HMGB1, high-mobility group box 1; HVA, homovanillic acid; IL-1β, interleukin-1β; IL-6, interleukin-6; LPO, lipid peroxidation; MAO, monoamine oxidase; MDA, malondialdehyde; MPTP, 1-methyl-4-phenyl-1, 2, 3, 6-tetrahydropyridine; NE, norepinephrine; RAGE, receptor for advanced glycation endproducts; ROS, reactive oxygen species; SDH, succinate dehydrogenase; SOD, superoxide dismutase; TAS, total antioxidant status; TH, tyrosine hydroxylase; TNF-α, tumor necrosis factor-α; TOS, total oxidant status; TPs, tongue protrusions; UPDRS, unified Parkinson’s disease rating scale; VCMs, vacuous chewing movements.

Terpenoids are a large class of natural organic compounds composed of isoprene units, widely distributed in plants, fungi, and marine organisms, with some synthesized by animals. They represent an important group of bioactive compounds in TCM.

### Myrtenol

Extracted from Taxus species, this monoterpenoid exhibits anxiolytic (148), anticancer (149), anti-inflammatory, and antioxidant (150) properties. In a reserpine-induced animal model of Parkinsonism, chronic myrtenol-treatment protected against olfactory sensibility loss, restored short-term memory and decreased motor impairments induced by reserpine. Moreover, this treatment prevented dopaminergic depletion and reduced the oxidative status index in the dorsal striatum (151).

### Menthol

As the primary component of peppermint oil, this monoterpenoid was shown to promote ambulation in ICR (Institute of Cancer Research) mice (152). This effect was potentiated by bupropion, but attenuated by the DA antagonists chlorpromazine, haloperidol, fluphenazine, spiperone, and SCH12679. Moreover, prior exposure to reserpine decreased sensitivity to the ability of menthol in promoting ambulation. The TH inhibitor *α*-methyl-*p*-tyrosine decreased subsequent sensitivity to the effects of menthol. These results suggest that DA is involved in the abilities of menthol to promote ambulation in mice (153). In support of this suggestion, a recent animal study reported that menthol pretreatment significantly reduced the rotational behavior in lipopolysaccharide (LPS)-exposed PD model rats, while protected dopaminergic neurons against inflammation mediated damage through the prevention of the microglial-mediated neuro-inflammatory response via the inhibition of the AKT, p65, JNK1/2 and ERK1/2 signaling pathways (154).

### Carvacrol

Carvacrol is a monoterpene and has been linked to neuroprotection in several animal models of neurodegeneration, including ischemia, epilepsy and traumatic neuronal injury. In the mouse hemiparkinsonian model, carvacrol significantly reduced the asymmetrical use of the forelimbs induced by unilateral 6-OHDA, while attenuated the loss of TH immunostaining both in the substantia nigra and the striatum (155). In 6-OHDA-stimulated rats, carvacrol improved the locomotor activity, catalepsy, akinesia, bradykinesia, and motor coordination while increased GSH content and reduced MDA level (156). In a model of progressive parkinsonism induced by reserpine, carvacrol prevented impairments in motor (catalepsy and VCMs) and the decrease in TH immunostaining in the substantia nigra pars compact and dorsal striatum of rats (157).

### Glycyrrhizic acid

GA is a triterpenoid from *glycyrrhiza glabra*, renowned for its antioxidant, anti-apoptotic, and neuroprotective effects. In a mouse model of PD induced by sub-acute administration of MPTP, glycyrrhizin suppressed MPTP-induced high-mobility group box 1 (HMGB1) and receptor for advanced glycation endproducts (RAGE) upregulation while reducing MPTP-induced dopaminergic cell death in a dose dependent manner (158). In haloperidol-treated rats, GA reduced VCMs, TPs, and FJs, enhanced locomotion, upregulated GSH and SOD, and lowered MDA while activating the p-PI3K/p-Akt/Nrf2 pathway in the striatum. In the *in vitro* experiment, it diminished ROS and boosted viability in haloperidol-exposed SH-SY5Y cells, corroborating its neuroprotection (159). In PD mice induced by MPTP, GA significantly improved motor function and alleviated gait disturbances while attenuated pathological damage to dopaminergic neurons in the mouse brain (160).

### Oleanolic acid

OA is an oleanane-type triterpenoid with anti-aging and neuroprotective properties. In rats treated with 6-OHDA, forelimb use asymmetry was ameliorated by treatment with OA 7 days pre- and 1 day post-lesion. In the cylinder test, rats treated with OA used the forelimb contralateral to the lesioned hemisphere significantly more. Rats treated with OA 7 days pre- and 1 day post-lesion had more DA in the striatum than the non-treated or the 7 days after lesion rats (161). In a haloperidol-induced mouse EPS model, OA normalized EPS and cognitive deficits of the subjects. In addition, OA normalized levels of DA and Ach in the striatum which were increased by haloperidol. Moreover, the increased phosphorylated protein kinase A (PKA), extracellular signal regulated kinase (ERK) and cAMP response element binding protein (CREB) levels and c-FOS expression level induced by haloperidol were significantly decreased by OA in the striatum (162). In rotenone-treated mice, OA at all doses alleviated the core symptoms of PD-related chronic unpredictable stress, and improved motor and non-motor function. Moreover, OA therapy significantly lowered IL-1β, TNF-α, IL-6, oxidative stress, and elevated NE, DA, and 5-HT levels in the mouse brain (163). In a MPTP-induced mouse model of PD, pre-treatment with OA prevented PD-like motor symptoms, reduced neuronal death in the substantia nigra, and mitigated striatal neurodegeneration. Post-treatment with OA not only reduced these effects but also increased Bcl-2 and DJ-1 levels in the substantia nigra and striatum (164). DJ-1 is a protein encoded by the *PARK7* gene and serves as a vital redox-sensitive sensor and “guardian” against cellular stress.

### Lycopene

Lycopene is a tetraterpenoid in tomatoes, exhibiting neuroprotective and anti-inflammatory activities. In 2002, Suganuma et al. reported that a 4-week ingestion of the experimental diet containing 20% (w/w) lyophilized tomato powders before MPTP treatment prevented a decrease in the DA level in the striatum of mouse brain, suggesting that the tomato ingestion might serve as a preventive against neurodegenerative diseases such as PD (165). Similarly, intake of tomato-enriched diet protected from 6-OHDA-induced decreases in levels of DA, DOPAC, and HVA in the striatum of rats (166). And lycopene administration to the rotenone treated mice increased the activities of SOD, GPx and CAT in substantia nigra and right striatum while reversed the cognitive and motor deficits in the animals (167). In MPTP-treated mice, lycopene protected the depletion of striatal DA and its metabolites in a dose dependent manner, while attenuated oxidative stress and motor impairments (168). In haloperidol-treated rats, lycopene treatment combined with haloperidol significantly attenuated impairments in motor behaviors (vertical climbing, tactile placing, jumping, rotarod activity, grip strength, and narrow beam walking) and ameliorated relevant biological changes in biochemical (MDA, nitrite, and GSH), neurochemical (DA, 5-HT, NE, and 5-hydroxyindole-3-acetic acid (5-HIAA)), and neuroinflammatory markers (TNF, IL-1β, and IL-6) (169).

### Crocin

Crocin is a water-soluble carotenoid component with biological activity in *saffron*, belonging to the terpenoid glycoside derivatives. It has functions such as scavenging ROS (170), anti-inflammatory (171), and antioxidant (172). In a rat model of EPS, pretreatment with crocin (10 mg/kg) modified haloperidol-induced VCMs, TPs, and abnormal movements (decreased locomotor and exploratory activity), while restored levels of MDA and GSH in the hippocampus, cortex and striatum of rats (173). In Wistar rats, malathion induced Parkinson-like behaviors as evidenced by decreased locomotor activity, muscular coordination deficit, and cataleptic immobilization. However, these behavioral changes were significantly prevented in the rats treated with malathion plus crocin. Moreover, the co-treatment of malathion and crocin effectively prevented the malathion-induced decreases in AChE and GSH, and increases in lipid peroxidation, IL-6, and TNFα (174). In an animal model of PD, crocin administration significantly improved the performance of the rotenone-treated rats in bar and grid tests as well as postural instability tests. At the cellular level, crocin increased levels of TH and DA, while decreased α-synuclein levels (175). In Cisplatin-treated Wistar rats, crocin substantially mitigated the deleterious effects of cisplatin on avoidance memory, explorative behavior, motor coordination, and balance of the animals. Crocin also effectively avoided the negative effects of cisplatin on MDA, GPx, and CAT in the hippocampus (176). In a most recent animal study, the combination of crocin and clavulanic acid (a β-lactamase inhibitor with significant neuroprotective potential) showed powerful neuroprotection as indicated by a significant increase in GSH, marked decreases in TNFα, IL1β, and MDA in the striatum of the haloperidol-treated rats, while improved TD-like behaviors (catalepsy and orofacial dyskinesia) in the animals (177).

In a mouse model of PD, MPTP treatment significantly increased the average time taken for mice to come down from the rod in the pole test, but decreased the average time spent by the mice in the hanging. These behavioral changes, however, were effectively prevented in the MPTP + crocin group of mice. Importantly, the MPTP-induced decrease in mean number of TH-positive neurons in the substantia nigro of the mouse brain was effectively prevented in the MPTP + crocin group of mice (178). Similarly, crocin prophylaxis significantly alleviated rotenone-induced behavioural alterations such as increased anxiety, diminished exploratory behaviour, decreased motor co-ordination, and grip strength in mice. Biochemical analysis in the striatum of mice indicated that crocin prophylaxis significantly decreased the rotenone caused oxidative damage including enhanced levels of ROS, hydroperoxides, and MDA (179).

## Therapeutic effects of other bioactive compounds on the APDs-induced EPS/EPS-like behaviors and neurobiological changes (**Table 10**)

**Table 10:** Therapeutic effects of other compounds on EPS/EPS-like bheaviors and the underlying neurobiological changes.

Chemical compounds	Subjects	Therapeutic effects	Neurobiological changes	References
Lauric acid	Haloperidol-treated rats	Performances in the rotarod and beam walking tests ↑	TNF-α↓, NFκB↓, IL-8↓, IL-4↑, MDA↓, NO↓, SOD↑	(180)
Valeric acid	Reserpine treated rats Rotemone treated rats	VCMs↓	/ TBARS↓, ROS↓, MDA↓, CAT↑, GSH↑, SOD↑, IL-6↓, TNF-α↓, NO↓, MMP-9↓, Microglia↓, Astrocytes↓, α-synuclein↓	(181) (182)
Caerulein	SCZ patients; Haloperidol-treated rats IDPN-treated rats	Orofacial dyskinesia↓, Catalepsy↓, Dyskinesia↓	DA release↓, DA receptors↑	(183) (186) (187)
L-theanine	Haloperidol-treated rats	VCMs↓, TPs↓, FJs↓	NO↓, LPO↓, SOD↑, GSH↑, CAT↑	(188) (189)
β-glucan	Plasma samples treated with haloperidol; TCDD-treated rats	/	TBARS↓, SOD↑, CAT↑, GPx↑, GSH↑, SOD↑	(190) (191)
Vitamin E	Haloperidol-treated rats Haloperidol-treated rats SCZ patients	/ Orofacial dyskinesia ↓ 28.3% of patients with TD showed modest improvement by vitamin E	BDNF↑ BAX↓, Bcl-2↑	(192) (16) (193)

BDNF, brain derived neurotrophic factor; CAT, catalase; DA, dopamine; GPx, glutathione peroxidase; GSH, glutathione; IL-4, interleukin-4; IL-6, interleukin-6; IL-8, interleukin-8; LPO, lipid peroxidation; MDA, malondialdehyde; MMP-9, matrix metalloproteinase 9; NFκB, nuclear factor-κB; NO, nitric oxide; ROS, reactive oxygen species; SOD, superoxide dismutase; TBARS, thiobarbituric acid reactive substances; TNF-α, tumor necrosis factor-α.

In addition to the four main categories (flavonoids, phenolic compounds, alkaloids, and terpenoids) of bioactive compounds as reviewed above, the other bioactive compounds including organic acids, peptides and amino acids, polysaccharides (*β*-glucan), and vitamins have also been studied for their potential in preventing and treating EPS.

### Organic acids

This category of bioactive compounds includes lauric acid and valeric acid. They are carboxyl-containing compounds widely distributed in the flowers, leaves, stems, fruits, and roots of medicinal plants. Their neuroprotective effects have gained increasing attention in recent years, providing new insights for EPS treatment. Introduced here are lauric acid and valeric acid.

Lauric acid is a medium chain fatty acid found in coconut oil, various trees, and animal fats. It is rapidly metabolized and easily ingested in humans. In a haloperidol-induced PD rat model, lauric acid improved behavioral performances of rats in the rotarod and beam walking tests, while attenuating the oxidative stress levels (MDA, NO, SOD) and changes in inflammatory cytokines (mRNA levels of TNF-α level, NF-κB, IL-8, IL-4) in the rat brain. These findings suggest that long-term intake of lauric acid-rich foods or supplements may mitigate neurotoxicity induced by antipsychotics thus improving outcomes of schizophrenia patients (180).

Valeric acid is a straight chain alkyl carboxylic acid found naturally in *Valeriana officinalis*. In previous preclinical *in vitro* and *in vivo* studies, valeric acid has been found to possess anti-cancer, anti-diabetic, antihypertensive, anti-inflammatory, and immuno-modulatory activities, and to affect molecular pathways related to various diseases such as Alzheimer’s disease, Parkinson’s disease, and epilepsy. In rats, valeric acid was found to reduce the intensity of reserpine-induced VCMs (181). In the rotenone-induced PD rat model, valeric acid prevented the rotenone-induced oxidative stress (decreases in CAT, GSH and SOD, and increase in MDA), diminished the rotenone-induced neuroinflammation as indicated by decreased IL-1ß, IL-6, TNF-α, NO, and matrix metalloproteinase 9 (MMP-9), along with inhibited microglia and astrocytes activation in the rat striatum, while protected dopaminergic neurons against rotenone toxicity (182).

### Peptides and amino acids

Introduced here are caerulein and L-Theanine.

Caerulein is *a*lso known as ceruletide. This bioactive peptide isolated from frog skin was shown to improve TD (particularly orofacial dyskinesia) in schizophrenia patients in an earlier clinical case report (183), whereas the other earlier clinical studies reported that there was no tendency for schizophrenia patients’ conditions to either improve or worsen during the course of ceruletide treatment (184, 185). Interestingly, ceruletide produced a relatively long-lasting inhibition of haloperidol-induced catalepsy in rats, while inhibited haloperidol (2 mg/kg)-induced increase in DA release from the rat striatum (186). In the iminodipropionitrile (IDPN)-treated rats, ceruletide improved the IDPN-induced dyskinetic movement, which paralleled changes in the DA receptors in the striatum of rats, suggesting that an up-regulation of DA receptors in the striatum corresponds with an improvement of dyskinesia in the IDPN-treated rats (187).

L-Theanine is the primary non-protein amino acid in tea leaves and has potent antioxidant and neuroprotective effects. In rats, L-Theanine treatment (100, 300 mg/kg orally for 35 days, starting 14 days before haloperidol injection) prevented most of the haloperidol-induced orofacial dyskinesia, while reduced the LPO production and enhanced the antioxidation power in the rat striatum (188). Similarly, L-Theanine (100, 300 mg/kg orally for 14 days, starting 10 days before reserpine injection) prevented most of the reserpine-induced orofacial dyskinesia, while reduced the reserpine-induced LPO production, increased the antioxidation power and catecholamines in the striatum, as well as reduced the levels of neuroinflammatory and apoptotic markers (189).

### β-glucan

β-glucan is a polysaccharide derived from the yeast cell walls of species such as *Saccharomyces cerevisiae*. In an earlier clinical study, Dietrich-Muszalska et al. (190) showed that β-glucan significantly decreased peroxidation level in human plasma treated with haloperidol. In a recent animal study, β-glucan ameliorated the 2, 3, 7, 8-tetrachlorodibenzo-p-dioxin (TCDD) toxicity in liver and brain tissue of rats by decreasing the TCDD-induced high levels of TBARS, but increasing GSH, SOD, CAT and GPx activities there (191).

### Vitamin E (tocopherol)

As a critical antioxidant, vitamin E was reported to restore BDNF levels in the prefrontal cortex, striatum, substantia nigra, and globus pallidus of haloperidol-treated rats (192). It also reduced pro-apoptotic BAX and increased anti-apoptotic Bcl-2 in these regions of rat brain, while alleviated chronic haloperidol-induced orofacial dyskinesia, validating its role as an effective EPS therapeutic (16). Although these preclinical studies encourage the use of tocopherol in the treatment of schizophrenia, the results reported in clinical studies are inconsistent. According to an earlier meta-analysis of published studies, a subgroup (28.3%) of patients with TD who were treated with vitamin E demonstrated modest improvement (193). However, a Cochrane Database Systematic Review concluded that vitamin E does not appear to improve the symptoms of TD in schizophrenia patients, although it may prevent the progression of existing TD (194). Further clinical studies are required to interpret these seemingly inconsistent clinical research results by identifying possible associating factors. Indeed, a recent clinical study reported significant differences in the variables of H_2_O_2_, ·OH, peroxidase, α-tocopherol, total antioxidant capacity, matrix metalloproteinase-9, and tissue inhibitor of metalloproteinases-1 across treatment-resistant schizophrenia, chronically medicated schizophrenia patients, and healthy controls (195).

## Neurobiological mechanisms underlying the therapeutic effects of phytomedicines on EPS/EPS-like behaviors

The APDs-induced EPS present with diverse manifestations and involve complex mechanisms, including dopaminergic dysfunction, oxidative stress, neuroinflammation, and mitochondrial dysfunction. In clinical practice, strategies such as discontinuing medication, taking anticholinergic drugs, or switching to SGA, are commonly used. However, challenges still remain to be resolved, as there are no specific medications to treat or prevent EPS. Fortunately, herbal medicines, due to their multi-target effects, fewer side effects, and abundant resources, show promising prospects for preventing and treating EPS induced by APDs. Of the molecular and neurobiological mechanisms involved in the therapeutic effects of phytomedicines, the follows deserve to be emphasized.

### Modulating functions of monoaminergic and cholinergic systems

As a main monoamine neurotransmitter, DA regulates motor neurons, spatial memory, reward circuits, and arousal. DA signaling is closely linked to neurological disorders such as PD, HD, TS, and attention deficit hyperactivity disorder (ADHD).

DA is synthesized in dopaminergic neurons in certain brain regions such as the substantia nigra and ventral tegmental area (VTA). DA in the striatum has at least two types of effects. It excites the GABA/dynorphin output via a predominance of D1-type receptors and inhibits the GABA/enkephalin output via D2-type receptors (196, 197). ACh interneurons in the striatum are thought to do the opposite of DA, i.e., inhibiting output cells with D1 receptors but exciting the GABA/enkephalin output (198). Prolonged blockade of DA receptors may disrupt the imbalance between dopaminergic inputs and cholinergic interneurons within the striatum. This balance is critical for maintaining normal motor function as indicated by the clinical practice that mAChR antagonists have efficacy in reducing motor symptoms in PD patients. But the efficacy is limited in their utility because of severe adverse effects (199, 200).

Many phytomedicines show ability to modulate DA and related neurotransmitters. For example, icariin (105) and myrtenol (151) upregulate TH thus enhancing DA synthesis. In the other studies, plumbago zeylanica root extract (76, 181) and quercetin (22, 23) inhibited COMT thus preventing excessive DA oxidation. Additionally, quercetin suppresses MAO thereby slowing DA metabolism. The other examples are *Hippophae rhamnoides* fruit extract, which upregulated DA and its metabolites (HVA, DOPAC), thereby reversing orofacial dyskinesia in TD rats (79). Furthermore, compounds like rice bran oil (56), naringin (25), and lycopene (169), enhanced 5-HT and NE activity, thereby bolstering monoaminergic resistance to antipsychotic toxicity.

Phytomedicines also target the cholinergic system. In EPS, Ach hyperactivity disrupts the DA-Ach equilibrium. Haloperidol-exposed animals showed higher AChE and BChE activity, but *Vitex negundo* leaf extract (72) and *Nigella sativa* seed oil (54, 55) inhibited these enzymes, thereby counteracting Ach hyperactivity and reducing VCMs/TPs.

### Alleviating oxidative stress

Oxidative stress refers to an imbalance between oxidation and antioxidation processes in cells, leading to increased ROS, which consequently triggers chain reactions that damage DNA, proteins, lipids and other biomolecules, ultimately resulting in biological dysfunction (201). The oxidative stress hypothesis is currently the most widely accepted pathogenesis mechanism for EPS. Related to this, many phytomedicines show antioxidative effects, exemplified by methanol extract of *Morus alba* leaves, vitexin, and curcumin. Of them, curcumin effectively alleviated the EPS phenotypes of VCMs, TPs, FJs, and stereotypic behaviors in rats exposed to haloperidol, while reversed the oxidative stress thus restoring DA, 5-HT, and NE turnover in the haloperidol-exposed rats (19). The *Morus alba* extract decreased vacuous chewing movements and tongue protrusions induced by haloperidol in rats, while attenuated haloperidol-induced lipid peroxidation and normalized levels of SOD, CAT, protein in comparison to the control group (60). Vitexin (a C-glycosylated flavone found in various medicinal herbs) effectively reduced haloperidol-induced orofacial dyskinesia, decreased oxidative stress, enhanced antioxidant capacity, and prevented mitochondrial dysfunction in the striatum of rats. But the protective effects of vitexin on the both behavioral and biochemical aspects were significantly reduced when trigonelline (an inhibitor of the Nrf2-mediated pathway) was administered (95).

### Restoring mitochondrial functions

Mitochondria play essential role in the catabolism of DA. In a state of elevated DA or increased DA oxidation exemplified by chronic or repeated administration of APDs, higher level of DA is toxic to mitochondria of neural cells in the brain (202). Consequently, mitochondrial defects aggravate DA elevation and lead to oxidative stress. This interaction between DA and mitochondrion has essential roles in the pathogenesis of PD and schizophrenia (203). Therefore, it is advocated to protect mitochondrial function in treating patients with schizophrenia by combination use of antioxidants and antipsychotics, instead of using antipsychotics only (204). In support of this advocation, when provided to schizophrenia patients affected by TD, Ginkgo biloba (at a dose of 240 mg/day) was effective in reducing AIMS scores (205). In another sample of patients with TD and schizophrenia, vitamin E was more effective than placebo at reducing the AIMS score along with an increase in blood SOD levels (206). The other two studies replicated the reduction in the AIMS score induced by vitamin E compared to the placebo (207, 208). A recent meta-analysis analyzed a total of 21 studies including 854 patients with TD and reported a decrease of 2.36 (95% CI = -3.27 to -1.45; P < 0.00001) in the AIMS score in the vitamin E treatment group, compared with the control group, suggesting vitamin E may offer a new avenue treatment for TD (209).

### Inhibiting neuroinflammation

As described above, chronic administration of APDs results in mitochondria dysfunction in neural cells of the brain, followed by oxidative stress and neuroinflammation, in which microglia, neurons, astrocytes, and peripheral immune cells work in hand with cytokines, chemokines, and the complement system to organize an inflammatory response. Activated microglia and astrocytes release proinflammatory cytokines (IL-1β, IL-2, IL-6, TNF-α etc.) (210) that damage brain tissue and increase blood-brain barrier (BBB) permeability (211). As such, neuroinflammation is considered an important factor in the progression and prognosis of EPS.

In line with the neuroinflammation mechanism of EPS, nigella sativa oil reduced EPS-like behaviors in haloperidol-treated rats while inhibiting astrogliosis in caudate and accumbens nuclei (54). In a recent animal study, vitexin mitigated haloperidol-induced orofacial dyskinesia in rats, while alleviating the haloperidol-induced high levels of IL-1β, IL-6, and TNF-α in the stratum through activation of the Nrf2 pathway (95). In a haloperidol-induced Parkinson rat model, MECP significantly decreased cataleptic scores and improved hanging and ladder climbing behaviors in haloperidol-treated rats, while decreasing mRNA levels of IL-1ß and TNF-α in the rat brain (62).

### Regulating the KEAP1/Nrf2 pathway

**Figure 1:**
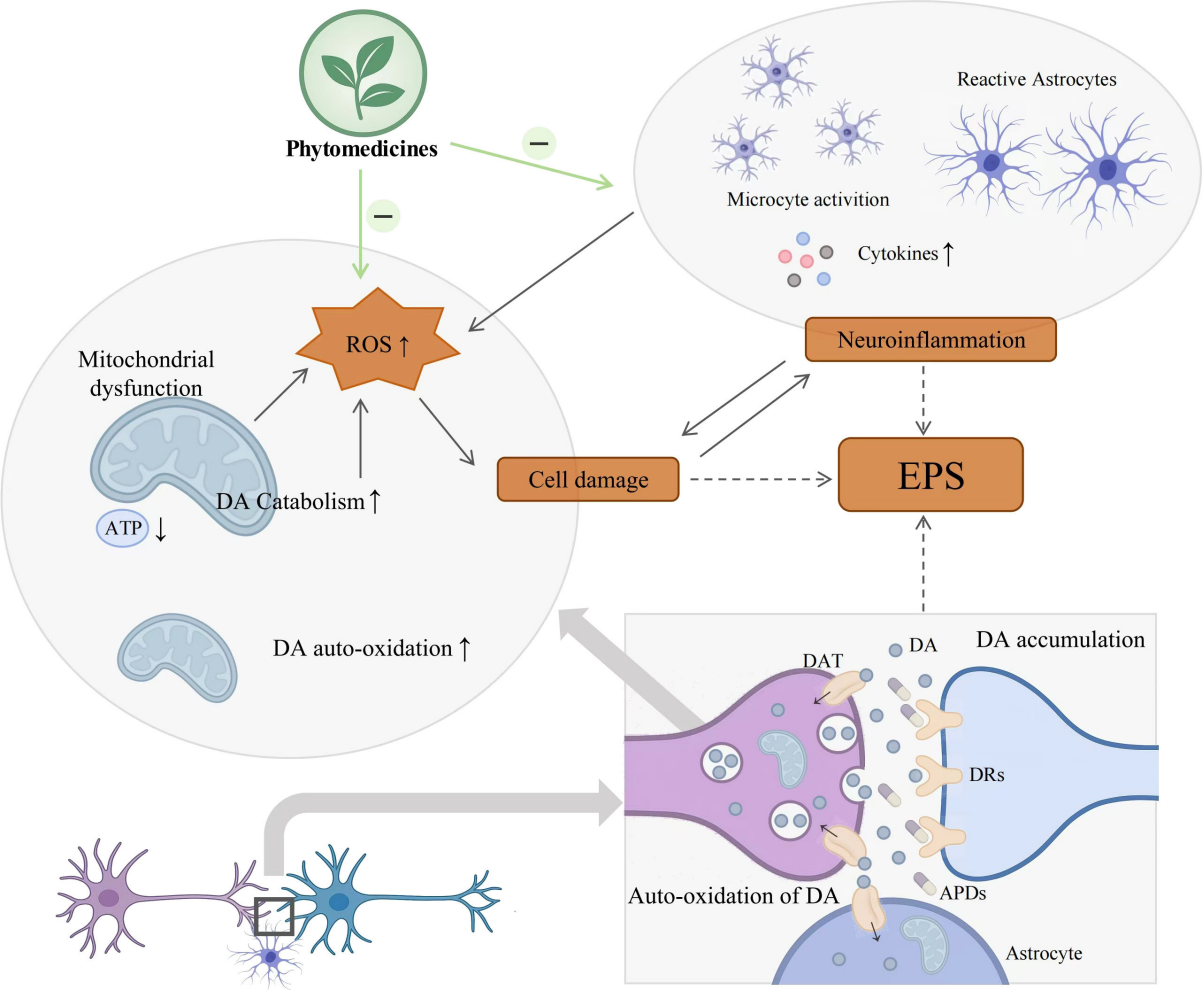
APDs block DA receptors thus increasing DA levels in synaptic clefts and presynaptic neurons. Consequently, high levels of DA auto-oxidation and catabolism produce more ROS, leading to mitochondrial dysfunction and cell damage, and even cell death of dopaminergic neurons in the substantia nigra, ultimately resulting in EPS. By anti-neuroinflammatory and antioxidative actions, phytomedicines protect neural cells and improve EPS.

The Keap1/Nrf2 pathway is a key regulator of antioxidant defense mechanisms. It regulates redox homeostasis and mitochondrial function, as well as plays a crucial role in modulating neuroinflammation (212–215). Under physiological conditions, Nrf2 binds to KEAP1. In the presence of ROS or under the condition of oxidative stress, Nrf2 dissociates from KEAP1 and translocates to the nucleus, where it activates the Nrf2/ARE pathway to regulate downstream targets like NAD(P)H quinone oxidoreductase-1 (NQO-1) and heme oxygenase-1 (HO-1) (216). Emerging data suggest that oxidative stress and neuroinflammation are major contributions to the pathogenesis of schizophrenia (217, 218). And patients with schizophrenia exhibited underexpression of KEAP1 and increased MDA concentrations, indicating enhanced lipid peroxidation and oxidative stress. Reduced GPX4 (glutathione peroxidase 4) activity and GSH levels were also observed (219). A recent animal study reported that geraniol (a natural aromatic compound found in plants such as lemongrass and citrus) showed neuroprotective and neurorestorative effects against schizophrenia-like symptoms by inhibiting oxidative stress and neuroinflammation in the mouse brain (220). In haloperidol-treated rats, GA reduced VCMs, TPs, and FJs, enhanced locomotion, upregulated GSH and SOD, and lowered MDA while activating the p-PI3K/p-Akt/Nrf2 pathway in the striatum as mentioned before (159). In mice exposed to cuprizone (a copper chelator toxic to mitochondria), baicalein prevented excessive activation of Nrf2 and its downstream antioxidant enzymes (HO-1, NQO1, and SOD2), thereby maintaining the signaling pathway at normal levels and promoting the recovery of animal motor and cognitive impairments (221). Taken together, all the above studies indicate the presence of dysfunctional Keap1/Nrf2 pathway in schizophrenia patients and suggest that this pathway may be targeted by some phytomedicines. It is this molecular mechanism that enables these phytomedicines to exert their antioxidant and anti-inflammatory effects, thereby exerting therapeutic effects on schizophrenia symptoms and EPS (**Figure 1**).

## Concluding remarks

Although APDs-induced EPS has a definite etiology as implied by the term, its clinical phenotype varies greatly at different stages of APDs treatment. The first three phenotypes of the APDs-induced EPS are collectively termed early-onset movement disorders, whereas TD typically emerges after three months of antipsychotic treatment and represents the most severe manifestation. Theoretically, the best treatment for the APDs-induced EPS is to withdraw antipsychotics in the first place. In clinical practice, however, avoiding antipsychotics is impossible. Even if managed through SGAs substitution under medical supervision, EPS cannot be completely prevented, because long-term use of SGAs also induces EPS, although the incidence rate is relatively low (14). Furthermore, anticholinergics, which are usually considered first-line treatment for patients with severe tremor, have been reported to increase the risk of a long-term cognitive decline such as dementia (222, 223).

Almost all APDs bind to DA receptors. Through this action, APDs exert their therapeutic effects on the positive symptoms in patients with schizophrenia. But the other side of the coin did not get enough attention. That is, APDs induce EPS via blocking DA receptors too. Specifically, long-term blockade of DA receptors leads to elevation of DA levels in the synaptic clefts between presynaptic terminals and postsynaptic neurons in the striatum. Consequently, the amount of DA transported to the cytoplasm of local astrocytes and oligodendrocytes in the striatum, as well as that of presynaptic neurons in the substantia nigra, increases. Under conditions of elevated DA levels, the auto-oxidation of DA in the synaptic space and the catabolism of DA in mitochondria of striatal glia cells and presynaptic neurons in the substantia nigra increase, ultimately leading to the accumulation of ROS in these locations. It is this persistent ROS that leads to neurodegeneration in the striatum and substantia nigra (224, 225).

As reviewed in this article, phytomedicines have shown great potential in intervening the APDs-induced EPS through their antioxidant and/or anti-inflammatory effects. Unfortunately, there are not many clinical medicine studies in this area yet. Therefore, we are unable to conduct a meta-analysis based on a large number of high-quality clinical studies of any one of phytomedicines. It is this limitation that leads to the imbalance between preclinical and clinical data in this manuscript. Preclinical studies involve whole herb formulations, herbal extracts, and isolated compounds, resulting in heterogeneity in intervention measures. Therefore, we are unable to provide practical recommendations on important issues such as dose variability, bioavailability, and herbal drug interactions in the clinical applications. The limited clinical research does not allow us to discuss the other scientific issues of study limitations, bias, confounders, sample sizes, and so on.

Pointing out the above limitations should encourage rather than limit clinical and translational medicine research in this field. Indeed, many of phytomedicines are widely used in clinical practice for the treatment of some physical diseases due to their efficacy and safety. For example, rice bran oil has been used for patients with hyperlipidemia (226, 227) and diabetes (228), approving its safety in clinical practice. And the efficacy and safety of EGb as an adjunct therapy in chronic schizophrenia have been reviewed in a systematic review of randomized, double-blind, placebo-controlled studies with meta-analysis, which showed no distinguishable difference between EGb and placebo group in mean total scores of TESS or a Rating Scale for Extrapyramidal Side Effects (229). Last but not least, the safety of many phytomedicines reviewed in this article, including berberine, betaine, caffeine and theophylline, curcumin, epicatechin, hesperetin, isoflavones, murrayanine, and naringin, has been approved, as demonstrated by the daily consumption of the fruits and plants rich in these compounds by humans. Pointing out these is not to say that there is no need to test the safety of these phytomedicines, but to encourage further preclinical and clinical studies aiming to increase more options for treating APD-induced EPS after further confirming their safety and efficacy.

## Glossary

3-NP: 3-nitropropionic acid
5-HT: serotonin
5-HIAA: 5-hydroxyindole-3-acetic acid
6-OHDA: 6-hydroxydopamine
ACh: acetylcholine
AChE: acetylcholinesterase
ADHD: attention deficit hyperactivity disorder
AIMS: abnormal involuntary movement scale
APDs: antipsychotic drugs
BBB: blood-brain barrier
Bcl-2: B-cell lymphoma 2
BDNF: brain-derived neurotrophic factor
BPRS: brief psychiatric rating Scale
BT: betaine
CAT: catalase
CBD: cannabidiol
CREB: cAMP response element binding protein
DA: dopamine
DAT: dopamine transporter
DIP: drug-induced parkinsonism
DOPAC: dihydroxyphenylacetic acid
DRs: dopamine receptors
EAF-HP: ethyl acetate fraction of harpagophytum procumbens
EGb: ethanol extract of ginkgo biloba
EGCG: epigallocatechin gallate
EPS: extrapyramideval syndrome
ERK: extracellular signal regulated kinase
ERP: ethanol extract of rubia peregrina
FJs: facial jerkings
GA: Glycyrrhizic acid
GABA: gamma-aminobutyric acid
GLRA1: glycine receptor, alpha 1 subunit
GPx: glutathione peroxidase
GR: glutathione reductase
GSH: glutathione
GST: glutathione-S-transferase
HD: huntington’s disease
H2O2: hydrogen peroxide
HMGB1: high-mobility group box 1
HO-1: heme oxygenase-1
HP: harpagophytum procumbens
HVA: homovanillic acid
IDPN: iminodipropionitrile
IL-1β: interleukin-1β
iNOS: inducible nitric oxide synthase
IP: aqueous extract of ilex paraguariensis
KGE: ethanol extract of Korean ginseng
LDH: lactate dehydrogenase
LIMK1: LIM-kinase 1
LPO: lipid peroxidation
LPS: lipopolysaccharide
LT: L-theanine
MAE: methanol-extract of Morus alba
MAO: monoamine oxidase
MDA: malondialdehyde
MECP: methanol extract of Cucurbita pepo seeds
MEME: methanol extract of Myrica esculenta
MEMP: methanol extract of Mucuna pruriens
MK: murrayanine
MMP-9: matrix metalloproteinase 9
MPP+: toxicity of 1-methyl-4-phenylpyridinium
MPTP: 1-methyl-4-phenyl-1,2,3,6-tetrahydropyridine
NQO-1: NAD(P)H quinone oxidoreductase-1
NE: norepinephrine
NF-κB: nuclear factor-κB
NO: nitric oxide
Nrf2: nuclear factor erythroid 2-related factor 2
NT: neurofibrillary tangle
OA: oleanolic acid
p38-MAPK: p38 mitogen-activated protein kinase
PANSS: positive and negative syndrome scale
PD: parkinson’s disease
PKA: protein kinase A
PRL: prolactin
PZE: hydroalcoholic extract of plumbago zeylanica leaves
RAGE: receptor for advanced glycation endproducts
ROS: reactive oxygen species
SBT: aqueous extract of sea buckthorn
SDH: succinate dehydrogenase
SGAs: second-generation antipsychotics
SKT: shakuyaku-kanzo-to
SNCA: alpha-synuclein gene
SOD: superoxide dismutase
TCDD: 2,3,7,8-Tetrachlorodibenzo-p-dioxin
TCM: traditional chinese medicine
TD: tardive dyskinesia
TESS: treatment emergent symptom scale
TH: tyrosine hydroxylase
TLR4: the Toll-like receptor 4
TNF-α: tumor necrosis factor-α
TPs: tongue protrusions
TS: tourette syndrome
Val: valeric acid
VCMs: vacuous chewing movements
VDAC1: voltage-dependent anion channel I
VNL: hydroalcoholic extract of vitex negundo leaves
VTA: ventral tegmental area
VTX: vitexin
WDD: wendan decoction
WS: aqueous extract of withania somnifera
YKS: yokukansan
